# UFO: A tool for unifying biomedical ontology-based semantic similarity calculation, enrichment analysis and visualization

**DOI:** 10.1371/journal.pone.0235670

**Published:** 2020-07-09

**Authors:** Duc-Hau Le

**Affiliations:** 1 Department of Computational Biomedicine, Vingroup Big Data Institute, Hanoi, Vietnam; 2 School of Computer Science and Engineering, Thuyloi University, Hanoi, Vietnam; Brown University, UNITED STATES

## Abstract

**Background:**

Biomedical ontologies have been growing quickly and proven to be useful in many biomedical applications. Important applications of those data include estimating the functional similarity between ontology terms and between annotated biomedical entities, analyzing enrichment for a set of biomedical entities. Many semantic similarity calculation and enrichment analysis methods have been proposed for such applications. Also, a number of tools implementing the methods have been developed on different platforms. However, these tools have implemented a small number of the semantic similarity calculation and enrichment analysis methods for a certain type of biomedical ontology. Note that the methods can be applied to all types of biomedical ontologies. More importantly, each method can be dominant in different applications; thus, users have more choice with more number of methods implemented in tools. Also, more functions would facilitate their task with ontology.

**Results:**

In this study, we developed a Cytoscape app, named UFO, which unifies most of the semantic similarity measures for between-term and between-entity similarity calculation for all types of biomedical ontologies in OBO format. Based on the similarity calculation, UFO can calculate the similarity between two sets of entities and weigh imported entity networks as well as generate functional similarity networks. Besides, it can perform enrichment analysis of a set of entities by different methods. Moreover, UFO can visualize structural relationships between ontology terms, annotating relationships between entities and terms, and functional similarity between entities. Finally, we demonstrated the ability of UFO through some case studies on finding the best semantic similarity measures for assessing the similarity between human disease phenotypes, constructing biomedical entity functional similarity networks for predicting disease-associated biomarkers, and performing enrichment analysis on a set of similar phenotypes.

**Conclusions:**

Taken together, UFO is expected to be a tool where biomedical ontologies can be exploited for various biomedical applications.

**Availability:**

UFO is distributed as a Cytoscape app, and can be downloaded freely at Cytoscape App (http://apps.cytoscape.org/apps/ufo) for non-commercial use

## Introduction

A number of biomedical ontologies have been built [1] such as Gene Ontology (GO) [2], Human Phenotype Ontology (HPO) [3], and Disease Ontology (DO) [4]. These data have been proven to be useful in many biomedical applications because they are used to annotate biomedical entities [5] such as genes [6, 7], phenotypes [3]. The use of these data is mainly based on semantic similarity calculation between ontology terms and between the annotated biomedical entities as well as enrichment analysis on a set of biomedical entities. Many semantic similarity measures have been proposed for such tasks [8]. More specifically, these measures are often used to measure the functional similarity between biomedical entities to construct functional similarity networks. These networks are then used in some biomedical applications, such as the prediction of disease-associated biomarkers. For example, relying on the assumption that functionally similar biomarkers are associated with phenotypically similar diseases, GO and HPO were respectively used to build gene similarity and disease similarity networks for predicting disease-associated biomarkers (e.g., gene and non-coding RNAs) [9–15]. GO was also used to predict gene/protein functions [16–18]. In addition, the biomedical ontology data are also used for enrichment analysis [19, 20].

The biomedical ontology data and the semantic similarity measures become useful in biomedical researches since many tools, which calculate the similarity, perform enrichment analysis and visualize the ontology, have been introduced. These tools run on different platforms such as Cytoscape, R statistics, Python, and Web, but implement a similar set of semantic similarity measures. The limitation of most of the tools is that they implemented a small set of measures for only one type of biomedical ontology. Indeed, among existing tools, only SML-toolkit [21] was developed generalized for all types of biomedical ontology and with a large number of semantic similarity measures. Other tools were only implemented with few of the measures and for a specific type of ontology such as GO (e.g., GOSim [22], ClueGO [23] and GOToolBox [24]), DO (e.g., DOSim [25], DOSE [26] and FunDO [27]) and HPO (e.g., HPOSim [28] and Phenomizer [29]). Besides, only a few tools provide visualization functions for biomedical ontology and annotated entities such as Gorilla [20], Golorize [30], and g:Profiler [31]. Note that the semantic similarity measures can be applied to any type of biomedical ontologies since all biomedical ontologies are represented in the same structure (i.e., directed acyclic graph). More importantly, each measure can be dominant in different applications [8, 32]; thus, the more number of measures are implemented in tools, the more choice for users to select the right measures for their application.

To overcome these limitations of the previous tools, we developed a Cytoscape [33] app, named UFO, which unifies most of the semantic similarity measures and can be used for any biomedical ontology in OBO format and annotation data. Based on the measures, the similarity between ontology terms and between entities can be calculated. Also, UFO can perform enrichment analysis for an entity set, weigh an entity network, and calculate the similarity between two entity sets. Furthermore, by exploiting visualization functions of Cytoscape, UFO can visualize the relationships between ontology terms and annotated biomedical entities. Those could improve the understanding of the semantic relationship between terms, annotated functions of biomedical entities, and their semantic similarity. Abilities of UFO were demonstrated through constructing entity similarity networks for predicting disease-associated biomarkers. Besides, enrichment analysis was performed on a set of phenotypes belonging to the same phenotypic series to identify significantly enriched HPO terms. Moreover, an assessment of various semantic similarity measures on a huge number of disease phenotype pairs figured out the best measures for estimating the similarity between two disease phenotypes.

## Design and implementation

**Fig 1:**
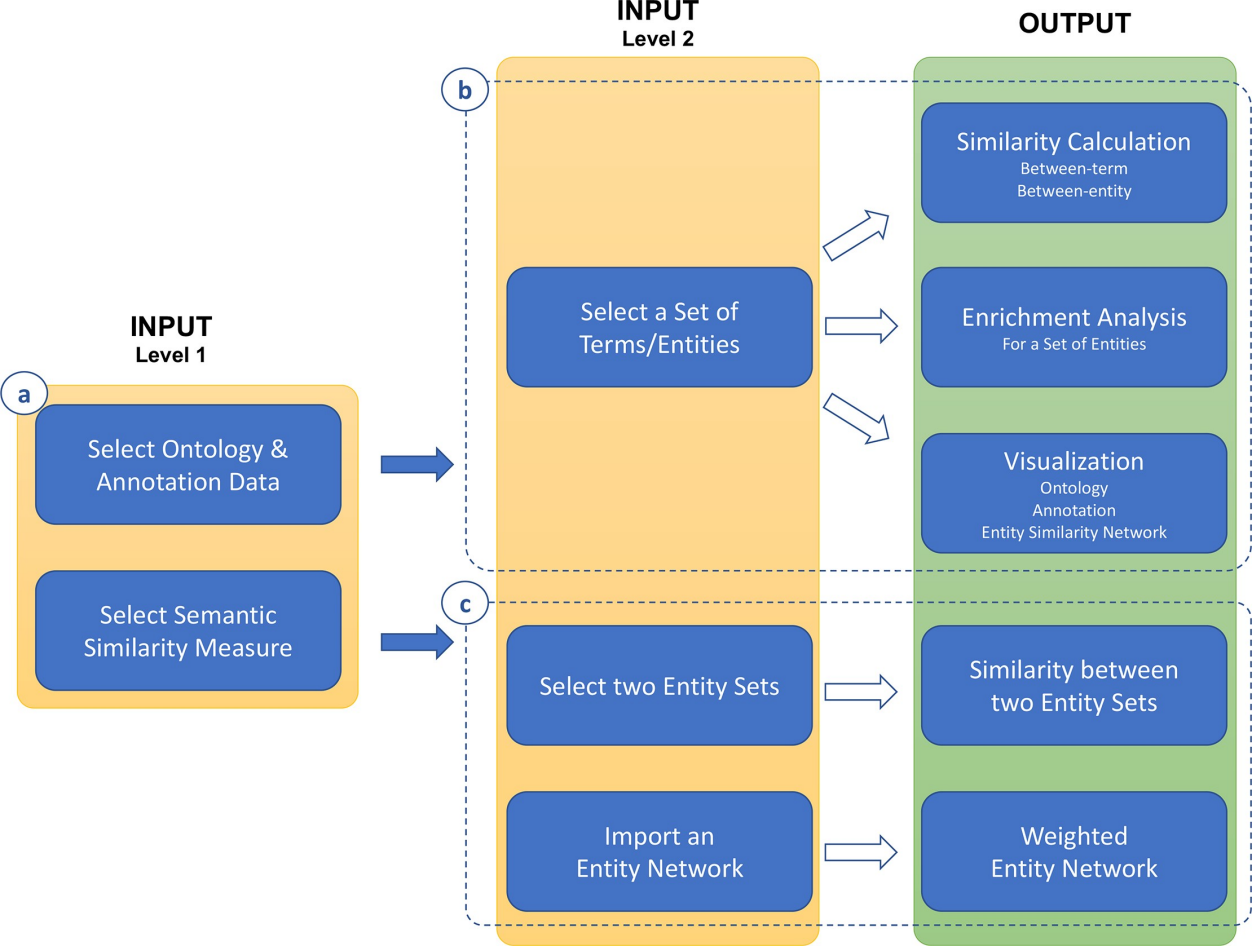
Implementation. **(a)** UFO is designed to work with any type of biomedical ontologies and various semantic similarity measures. **(b)** Main functions include similarity calculation, enrichment analysis, and visualization. **(c)** In addition, UFO can calculate the similarity between two entity sets and weigh imported entity networks.

UFO was designed to work with all types of biomedical ontologies in OBO format and various semantic similarity measures for similarity calculation, enrichment analysis, and visualization (Fig 1). For each task, the user can specify a biomedical ontology and annotation data, which can be either pre-installed with the app or user-imported. After that, depending on the task, a between-term similarity and a between-entity similarity measure must be selected (Fig 1(A)). Three main functions, including similarity calculation, enrichment analysis, and visualization, were implemented (Fig 1(B)). For a set of selected terms or entities, a similarity matrix containing the similarity between every pair of the selected terms or entities can be calculated. Then a directed acyclic graph of the selected terms, annotation (annotating terms for the selected entities or annotated entities of the selected terms), and functional relationships (i.e., similarities) between the selected entities can be visualized. Additionally, enrichment analysis can be performed for the selected entities and therefore help infer their functions, e.g., biological functions of a gene set. In addition to the main functions, UFO can calculate the similarity between two entity sets (this can be useful for e.g., comparing two gene sets, see more detail in S1 File) and weigh an imported entity network by the similarity between two interacting entities (Fig 1(C)). In the following sections, we briefly describe implemented semantic similarity measures and biomedical ontology/annotation data.

### Semantic similarity measures

The semantic similarity measures were defined for ontology terms and annotated entities, as reviewed in [8]. In UFO, we implemented eleven between-term similarity measures (including eight node-based (i.e., Resnik [34], Lin [35], JC [36], Rel [37], ResnikGraSM [38], LinGraSM [38], JCGraSM [38], RelGraSM [38], two edge-based (i.e., Wu2005 [39], and Yu2005 [40]), and one hybrid-based (i.e., Wang2007 [41]) measures) (Table 1). These between-term similarity measures can be used in four pairwise between-entity similarity measures to calculate the similarity between entities. In addition, we implemented seven group-wise between-entity similarity measures (including two vector-based (i.e., Cosine [42] and Kappa [43]) and five graph-based (i.e., TO [44], NTO [45], GIC [24, 46], UI [47], and LP [46]) measures) (Table 2).

**Table 1:** Between-term measures implemented in UFO.

*No*.	*Measure*	*Approach*	*Reference*
1	Resnik	Node-based	[34]
2	Lin	Node-based	[35]
3	JC	Node-based	[36]
4	Rel	Node-based	[37]
5	ResnikGraSM	Node-based	[38]
6	LinGraSM	Node-based	[38]
7	JCGraSM	Node-based	[38]
8	RelGraSM	Node-based	[38]
9	Wu2005	Edge-based	[39]
10	Yu2005	Edge-based	[40]
11	Wang2007	Hybrid-based	[41]

**Table 2:** Between-entity measures implemented in UFO.

*No*.	*Measure*	*Approach*	*Reference*
1	Avg	Pairwise-based	[48]
2	Max	Pairwise-based	[49, 50]
3	BMA	Pairwise-based	[38, 51, 52]
4	rcMax	Pairwise-based	[37]
5	TO	Groupwise-based	[44]
6	NTO	Groupwise-based	[45]
7	UI	Groupwise-based	[53]
8	LP	Groupwise-based	[53]
9	GIC	Groupwise-based	[47]
10	Cosine	Groupwise-based	[42]
11	Kappa	Groupwise-based	[43]

#### Between-term measures

Node-based measures were based on properties of terms themselves, their ancestors, or their descendants to compare. We implemented four basic information content (IC)-based measures, including Resnik [34], Lin [35], JC [36], and Rel [37]. These four measures are based on the most informative common ancestor (MICA), in which only the common ancestor with the highest IC is considered.

In addition to these measures, we implemented four variants of these measures; i.e., ResnikGraSM, LinGraSM, JCGraSM, and RelGraSM, which were based on the disjoint common ancestors (DCA), in which all disjoint common ancestors (the common ancestors that do not subsume any other common ancestor) were considered [38]. Edge-based measures are based mainly on counting the number of edges in the graph path between two terms. In particular, we implemented two edge-based methods, which are Wu2005 [39] and Yu2005 [40]. Finally, we implemented one hybrid measure, which was introduced in Wang [41]. Table 1 summarizes between-term measures implemented in UFO (See more detail about each measure in S1 File).

#### Between-entity measures

Pairwise-based methods measure the functional similarity between two biomedical entities by combining the semantic similarities between their terms. Each entity is represented by a set of annotating terms, then the semantic similarity is calculated between terms belonging to the two sets using one of the between-term measures. We implemented average (Avg) [48] and maximum (Max) [49, 50] techniques, which consider every pairwise combination of terms from the two sets. In addition, the other two methods considering only the best-matching pair for each term, i.e., best match average (BMA) [38, 51, 52] and a maximum of row and column scores (rcMax) [37], were implemented. For groupwise-based methods, we implemented two vector-based measures, i.e., Cosine [42], and Kappa [43] coefficients. In vector-based methods, the annotating term set of each entity is represented by a vector, then the semantic similarity between two entities is calculated based on the two representing vectors. In addition, we implemented five graph-based measures, i.e., TO [44], NTO [45], UI, LP [53], and GIC [47]. The graph-based methods are based on direct annotating terms and their ancestors, and the structure of the ontology graph. Table 2 summarizes between-entity measures implemented in UFO (See more detail about each measure in S1 File).

### Biomedical ontology and annotation data

There are a number of biomedical ontologies that have been built for biomedical entities at OBO Foundry [1]. Among them, ontologies for gene, disease, and human phenotype have been popularly used. Therefore, in this study, we pre-installed these ontologies and corresponding annotation data. Particularly, for GO, we collected GO term at OBO Foundry. The corresponding annotation data for genes were collected from NCBI FTP site (ftp.ncbi.nlm.nih.gov/gene/DATA/gene2go.gz). For HPO, we collected HPO terms and corresponding annotation data in the HPO database [3]. In addition, we also collected DO terms [4] and annotation data [7]. Other ontology and annotation data can also be loaded to UFO, in which ontology data is in a standard format.obo, meanwhile, the annotation data is as the following (for each line): EntityID<tab>OntologyTermID<tab>EvidenceCode (optional).

## Results

### Main functions

UFO was implemented with the main functions of similarity calculation, enrichment analysis, and visualization. Here, we briefly describe the main functions of UFO (See more detail in S2 File).

#### Similarity calculation

**Fig 2:**
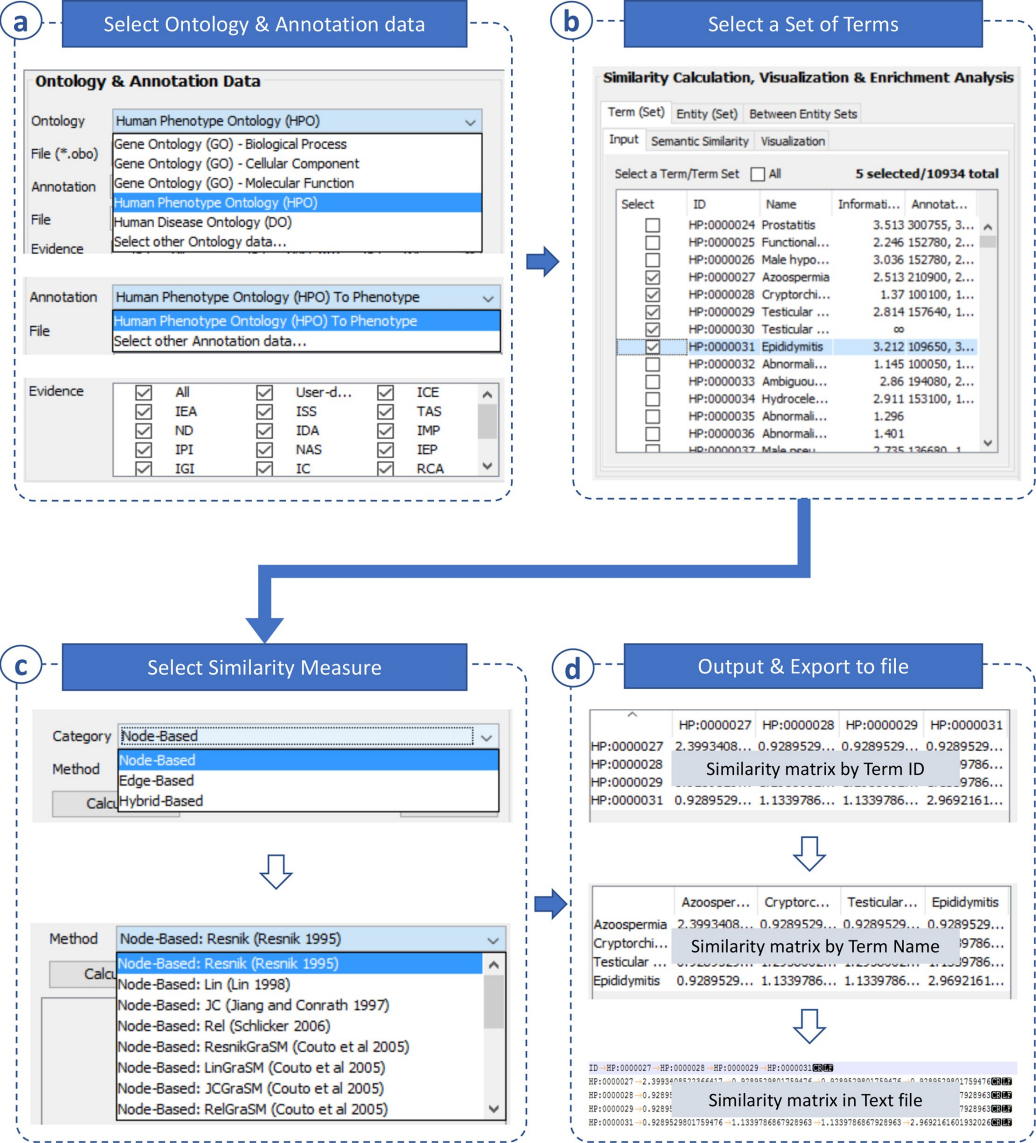
Between-term similarity. This function is completed in four following steps. **(a)** Select an ontology and annotation data as well as evidence of annotation. **(b)** Select a set of terms for similarity calculation. **(c)** Select a semantic similarity measure (choose a category of between-term similarity methods then a specific method in the category). **(d)** Calculate a similarity matrix for selected terms then export the result to a file.

**Fig 3:**
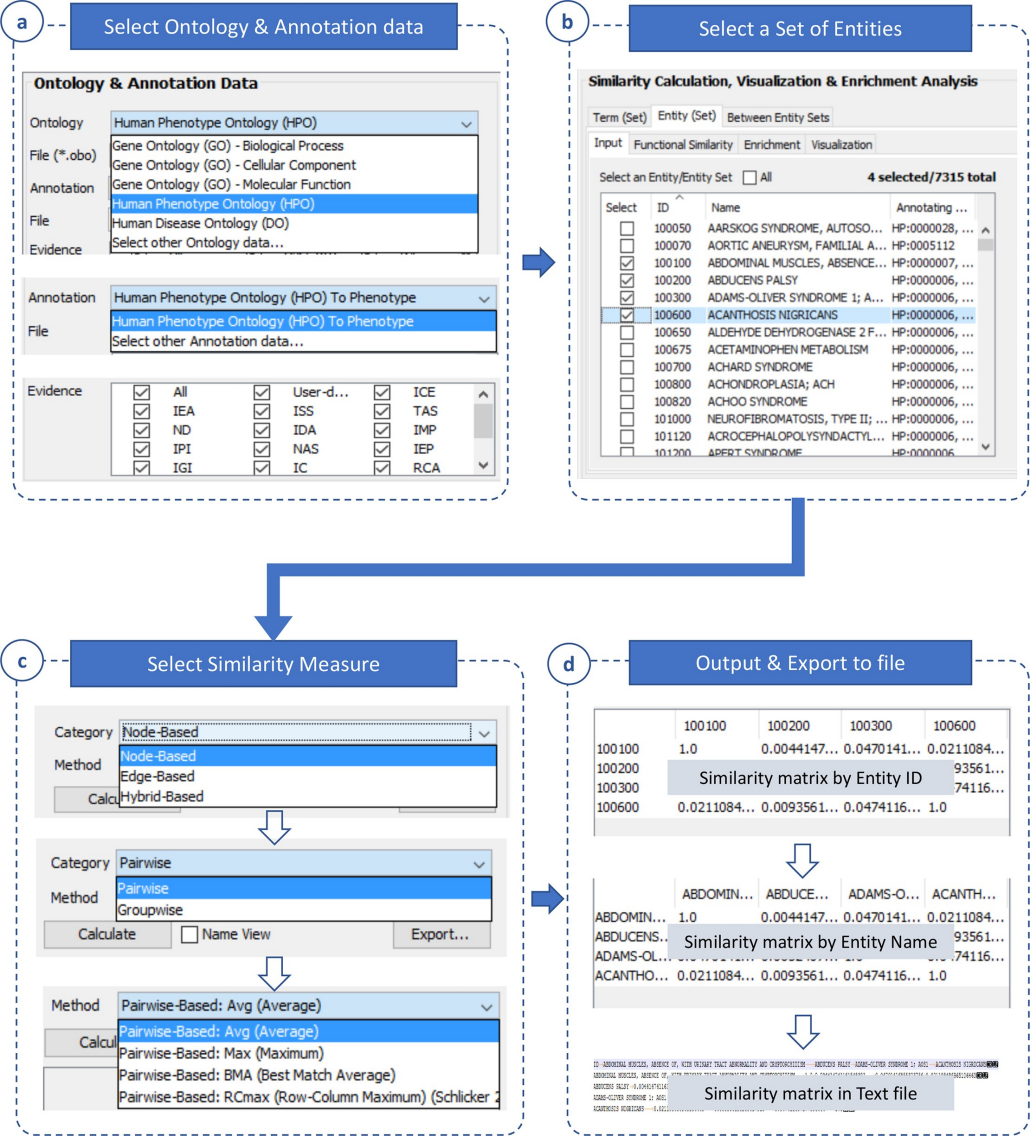
Between-entity similarity. This function is completed in four following steps. **(a)** Select an ontology and annotation data as well as evidence of annotation. **(b)** Select a set of entities for similarity calculation. **(c)** Select a semantic similarity measure (choose a specific between-term similarity method, then a specific between-entity similarity method). **(d)** Calculate a similarity matrix for selected entities then export the result to a file.

Similarities between ontology terms and between entities were represented by similarity matrices; then, they can be exported to file for further use. To this end, a set of terms or entities must be selected. Fig 2 shows a workflow for generating a similarity matrix of selected terms. Briefly, the similarity between two terms can be calculated using either a between-term node- or edge-based method (See more detail in S1 File). Similarly, Fig 3 shows a workflow for generating a similarity matrix of selected entities. In addition to the selection of a between-term similarity measure, a between-entity similarity measure must be specified (See more detail in S1 File). Then, these similarity matrices can be exported to text files for future use. Based on similarity calculation, UFO can calculate the similarity of two sets of entities and weigh imported entity networks as well as generate entity functional similarity networks.

#### Enrichment analysis

**Fig 4:**
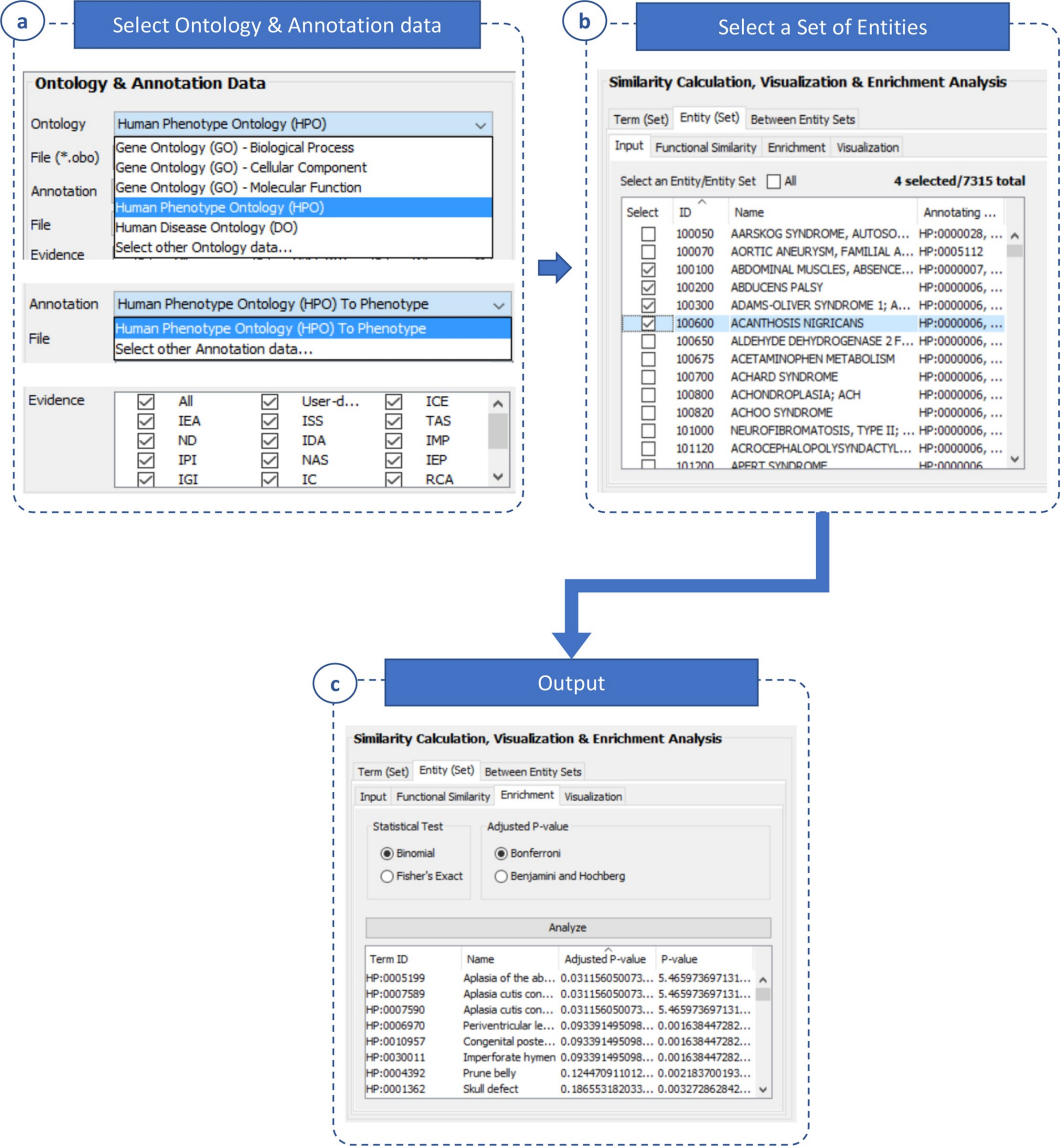
Enrichment analysis. This function is completed in four following steps. **(a)** Select an ontology and annotation data as well as evidence of annotation. **(b)** Select a set of entities for enrichment analysis. **(c)** Select a statistical test, and a multiple testing correction method then do the enrichment analysis for the selected entities.

UFO can perform enrichment analysis for a set of entities, and therefore help infer their common functions (Fig 4). Given an entity set (*S*_*e*_), the goal of enrichment analysis is to find statistically significant ontology terms enriching for the entity set. Given a term *t* annotating for at least one entity in the set, *t* is said to be statistically significant if there is a statistically significant overlap between the entity set and the set of entities annotated with term *t* in the corpus.

In UFO, two statistical tests were implemented, i.e., the Fisher’s exact test and the binomial test. A p-value indicating the probability of the null hypothesis (i.e., there is no significant association between *S*_*e*_ and *t*) will be obtained by a statistical test. When testing multiple hypotheses, the obtained p-values have to be adjusted in order to control the type I error (false positive) rate [54]; thus we also implemented the two multiple testing correction methods in UFO, i.e., Bonferroni [55], and Benjamini and Hochberg correction [56] (See S1 File for more detail about the statistical tests and the multiple testing correction methods).

After applying a multiple testing correction method, an adjusted p-value will be obtained for each term *t*. The p-value represents the probability of the null hypothesis; thus, the smaller p-value is the less likely that the association between *S*_*e*_ and *t* is random. In enrichment analysis, the adjusted p-value ≤0.05 indicates the association is statistically significant.

#### Visualization

**Fig 5:**
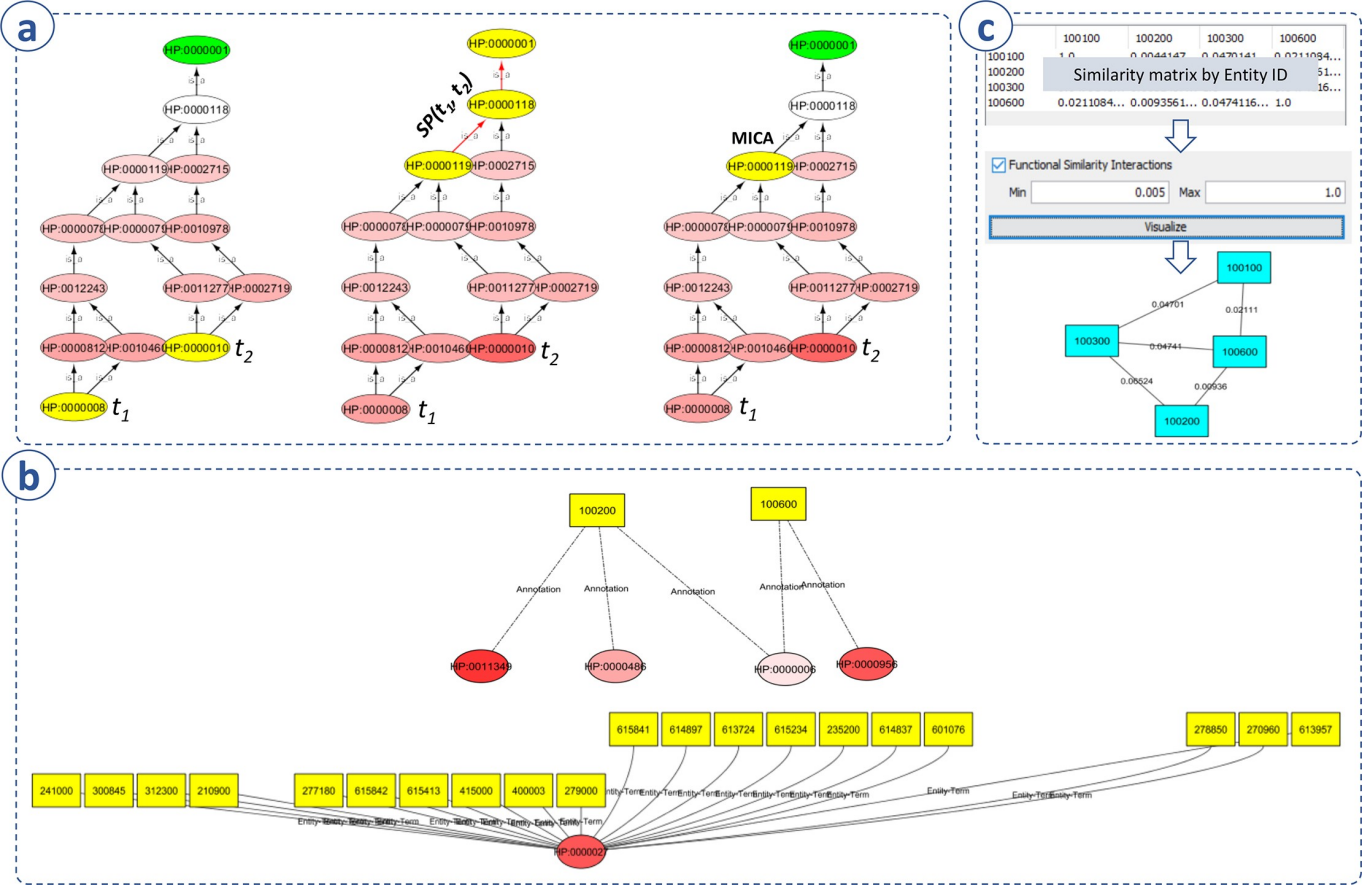
Visualization. **(a)** Selected terms can be visualized in a directed acyclic graph with a root term (green node). Color intensity is proportional to the information content (IC) (the higher IC correlated with a stronger red color). In addition, a shared path (SP) to the root term and shared ancestors of the selected terms are also indicated. Also, the most informative common ancestor (MICA) is specified. **(b)** When a set of entities is selected, UFO will visualize their annotating terms (upper panel). In contrast, when a set of terms is selected, UFO will visualize their annotated entities (lower panel). **(c)** After calculating the similarity matrix for a set of entities, the user can set thresholds (Min, Max) to create an entity similarity network from the similarity matrix and visualize it.

To facilitate the understanding of structural relationships between ontology terms, functional relationships between entities and terms (i.e., annotation), and between entities, we provided functions to visualize these relationships (Fig 5). More specifically, the relationship among selected terms was visualized in a directed acyclic graph, and with their ancestors and descendants. Their shortest path (SP) to the root term and shared ancestors were also indicated. In addition, the similarity between entities can be visualized in the form of a similarity network where interaction can be defined with pre-set thresholds of similarity degree.

### Comparison with other tools

We investigated 33 tools designed for biomedical ontology, including 23 tools for GO, three tools for DO, six tools for HPO, and one tool generalized for biomedical ontology. For GO, a total of 14 semantic similarity tools were also comprehensively reviewed in [57]. In this study, we additionally investigated nine tools including GOSemSim [58], Golorize [30], ClueGO [23], BiNGO [59], SimTrek [60], AmiGO [61], GOrilla [20], g:Profiler [31], and DynGO [62]. Besides, six HPO-based tools, including HPOSim [28], Phenomizer, OWLSim, PhenoDigm, PhenomeNET, and OntoSIML, were compared in [28]. For DO-based semantic similarity tools, we examined three tools, i.e., DOSim [25], DOSE [26], and FunDO [27]. The usability of the ontology tools is mainly dependent on the platform they were developed and the functions they provided. Thus, in this section, we compared UFO with the 33 tools in terms of their platform and functions (See more in S1 Table).

The tools were developed and run on different platforms (i.e., four Cytoscape plugins/apps, one Java package, eight R packages, two Python packages, 16 web-based tools, and two standalone ones). Each platform has its own advantages and disadvantages. For tools developed as packages in Java, R, and Python, they are convenient when integrated with other analysis pipelines, but dependent on such platforms. In addition, users are often required programming ability to use the tools. Meanwhile, web-based tools do not require programming ability but may limit users to use pre-installed ontology data. As the other four Cytoscape plugins/apps, UFO can also exploit the built-in integration functions of the platform. Indeed, biomedical entities such as genes/proteins in UFO can be easily annotated with pathways and protein complexes. In addition, ontology and annotation data can be freely provided by users. Moreover, entity networks (e.g., gene similarity network and phenotype similarity network) constructed by UFO can be directly integrated as inputs with other Cytoscape apps such as GPEC [10] and HGPEC [13] for predicting disease-associated genes. It should be noted that Cytoscape is nowadays a popular platform for developing apps for various biomedical problems [63].

The developed functions of the tools such as the semantic similarity calculation (i.e., between terms, between entities, between entity sets and weighting entity network), the enrichment analysis, and visualization are all directly related to the usability of the tools. Most of the tools provided the enrichment analysis and the semantic similarity calculation functions but with different sets of between-term and between-entity measures. The number of semantic measures implemented in UFO and SML-toolkit [21] is comparable and larger than any other tool. In addition to the enrichment analysis, some tools such as Golorize [30], BiNGO [59], AmiGO [61], GOrilla [20], g:Profiler [31], and DynGO [62] provided visualization functions, but only for directed acyclic graph and annotation of ontology; meanwhile, the visualization functions are not available in SML-toolkit. As a Cytoscape app, UFO can also exploit the built-in visualization functions of the platform. Indeed, the ontology graph, the relationship between terms and annotated entities, and the functional similarity between biomedical entities can be easily visualized by UFO. Based on the visualization, the complex relationship among ontology terms and biomedical entities is more interpretable. Finally, all tools are designed for a specific type of biomedical ontology such as GO, DO, and HPO, except UFO and SML-toolkit, thus they have limited their usability in such biomedical ontology-specific application.

### Case studies

We previously assessed human disease phenotype similarity based on HPO using a large set of similarity measures [64] using UFO. The results can provide an overall assessment and guidelines for future studies that need to choose the most approximate semantic similarity method to assess the phenotypic similarity between diseases. In addition, we employed UFO for constructing gene similarity networks, protein complex similarity network, and disease similarity networks using GO, HPO, and DO for predicting disease-associated genes [11, 65], protein complexes [66], and lncRNAs [14]. Besides showing applications of UFO in our previous studies, we additionally demonstrated its ability to find significantly enriched HPO terms for a set of similar phenotypes. Here, we shortly described how UFO was used for these applications.

#### Assessing human disease phenotype similarity based on HPO

Comparing different similarity measures could help researchers choose the most appropriate measure for their biological application. Mazandu *et al* [32] conducted a performance evaluation of a number of different functional similarity measures using different types of biological ontology to infer the best functional similarity measure for each semantic similarity approach. In the study [64], we collected 4,295 phenotypes with annotations from the HPO database [3] and calculated the similarity for all pairs of phenotypes using 47 between-entity similarity measures. This resulted in similarities for 9,221,365 pairs of phenotypes. To assess the performance of each between-entity similarity measure, we compared the similarity of these pairs with those collected from MimMiner [67], which has been popularly used in many studies, using Pearson and Spearman correlation coefficients. Simulation results showed that, for pairwise-based methods, the largest correlation coefficient was 0.58 for BMA and Max pairwise between-entity similarity with Rel and Wu2005 between-term measures, respectively [64]. For groupwise-based methods, best measures were LP between-entity for both correlation methods and TO between-entity method for Spearman correlation [64] (See more detail in S2 File).

#### Construct gene and protein complex similarity networks using GO for predicting disease-associated genes and protein complexes

**Fig 6:**
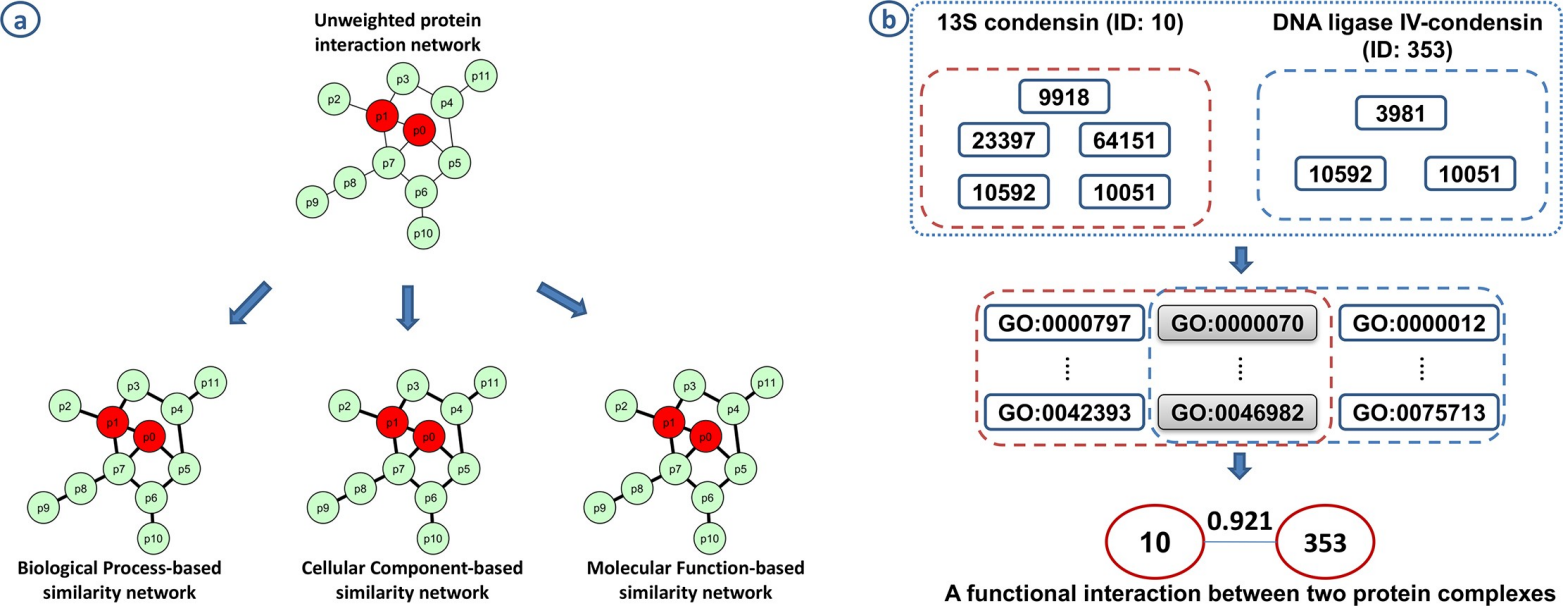
Construct gene and protein complex similarity network using GO. **(a)** Weighting protein interaction network using GO. Three GO subtypes (i.e., biological process, cellular component, and molecular function) were used to construct three weighted protein interaction networks from the unweighted one using a pairwise between-entity similarity method. **(b)** A functional similarity interaction between two protein complexes was created based on shared GO terms, which are used to annotate protein elements in protein complexes.

In the study [11], we used GO-based similarity to weigh a physical protein interaction network to create gene similarity networks using three types of gene ontology, i.e., biological process, cellular component, and molecular function using semantic similarity measures (Fig 6(A)). Then, these networks were used to predict disease-associated genes [11]. Particularly, each protein in the physical protein interaction network was annotated with GO terms. Then, the similarity between two proteins in an interaction was calculated using a pairwise between-entity measure. The similarity was then assigned as a weight for each protein interaction in the physical network. Finally, those weighted networks were used to predict novel disease-associated genes. In another study [66], the GO was also used to estimate the similarity between two protein complexes (Fig 6(B)), then to construct a functional similarity protein complex network. In particular, protein elements in a protein complex were annotated with GO terms. Then, the functional similarity between two protein complexes was assessed by shared GO terms. Finally, this network was used to predict novel disease-associated protein complexes.

#### Construct disease similarity network using HPO and DO for predicting disease-associated genes and lncRNAs

**Fig 7:**
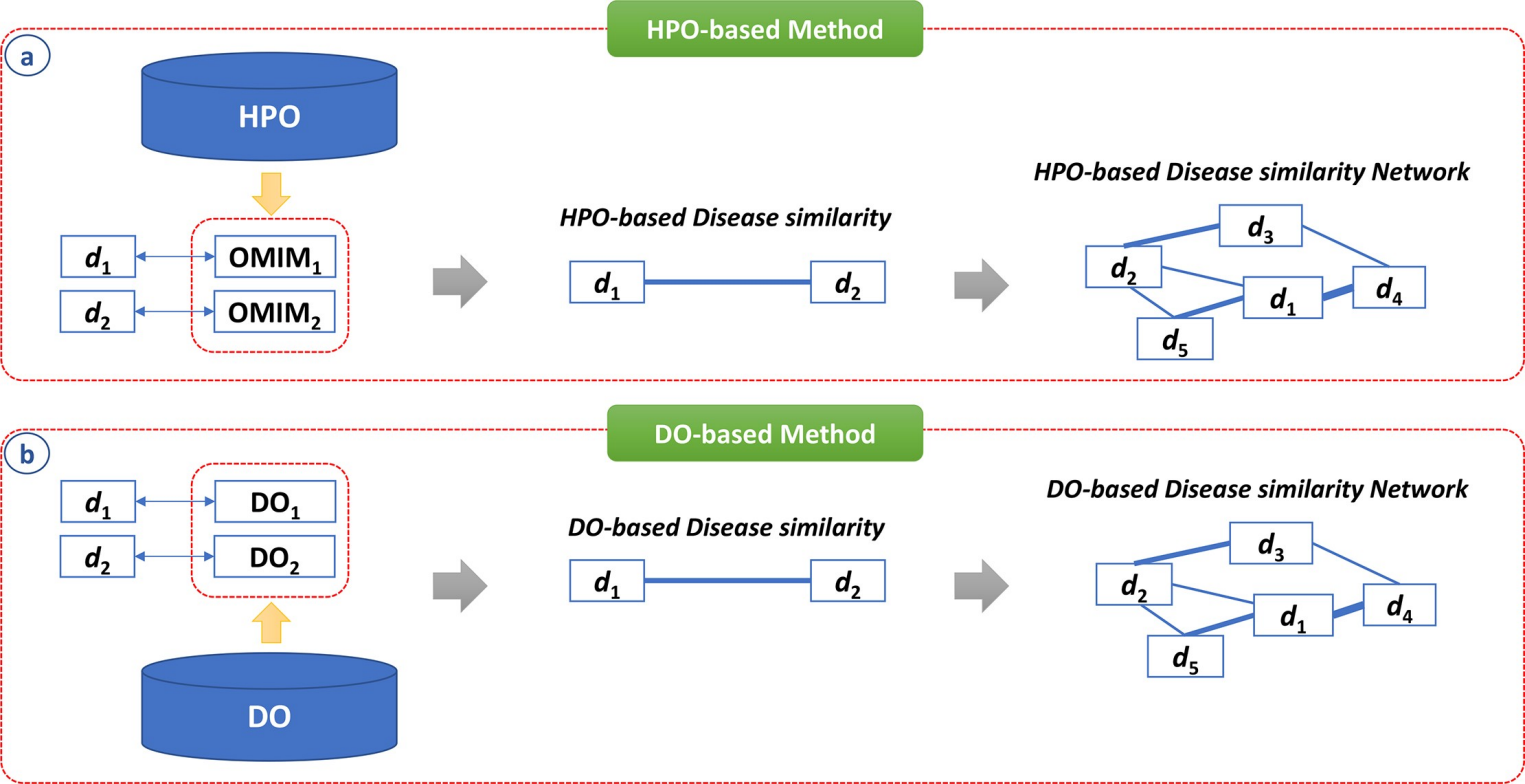
Construct disease similarity network using HPO and DO. **(a)** Diseases were mapped with OMIM ID, then the semantic similarity for every pair of OMIM IDs was calculated using annotating HPO terms. **(b)** Diseases were mapped with DO terms, then the semantic similarity for every pair of DO terms was calculated.

In addition to the construction of gene similarity networks using GO, we built a disease similarity network using HPO to improve the prediction of disease-associated genes [65]. Specifically, we showed that the HPO-based disease similarity network provides better prediction performance compared to the OMIM-based disease similarity network. In more detail, diseases were mapped with OMIM IDs, then the semantic similarity for every pair of OMIM IDs was calculated using annotating HPO terms (Fig 7(A)). In addition to HPO, the DO was also used to construct the disease similarity network for predicting disease-associated lncRNAs [14]. In the study, diseases were mapped with DO terms, then the semantic similarity for every pair of DO terms was calculated (Fig 7(B)).

#### Enrichment analysis with HPO

**Fig 8:**
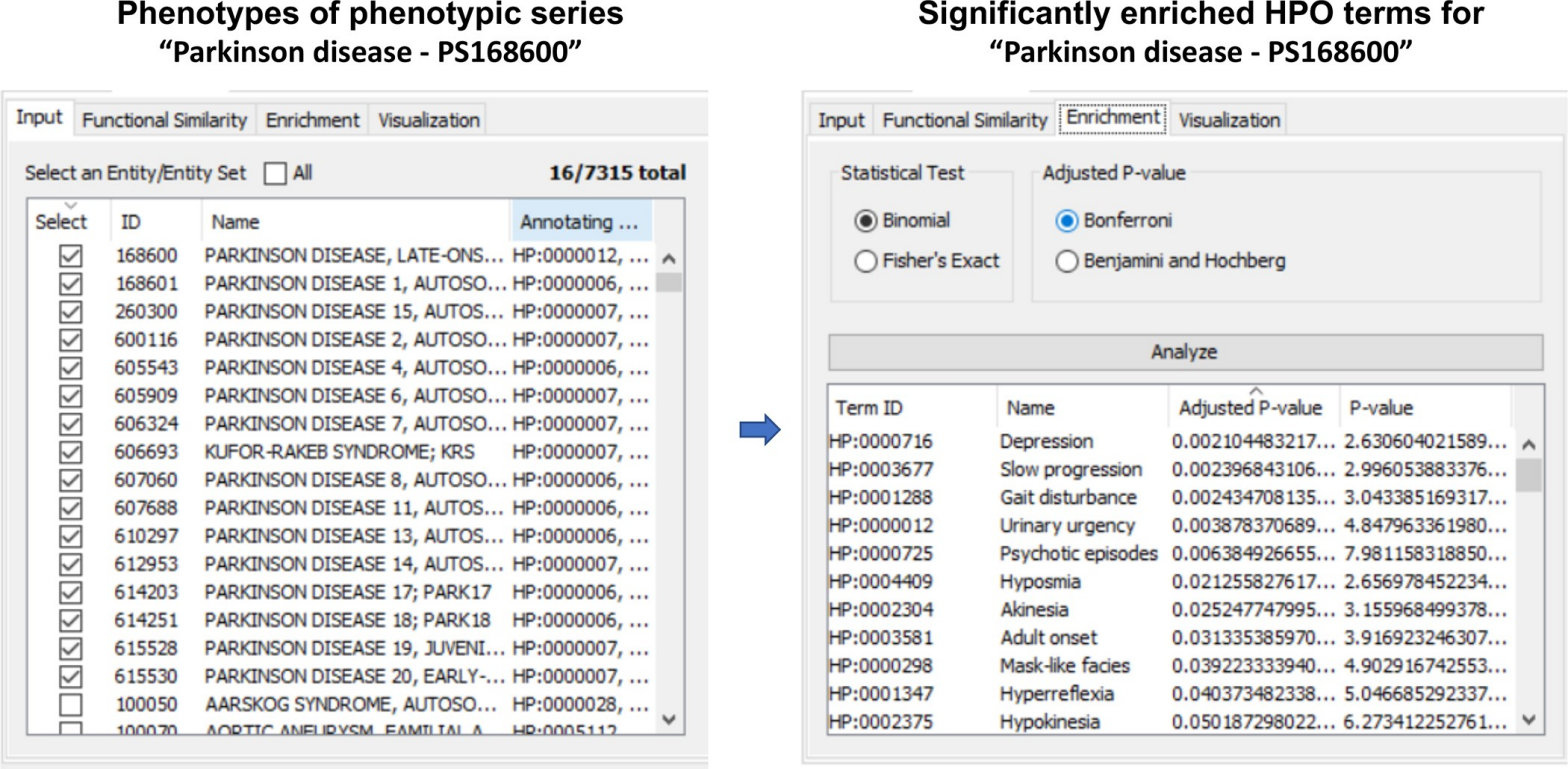
Enrichment analysis with HPO. Enrichment analysis for a set of 16 phenotypes of phenotypic series “Parkinson disease–PS168600”. A total of ten HPO terms significantly enriches for the set.

In this case study, we performed enrichment analysis for phenotypic series from OMIM (https://www.omim.org/phenotypicSeriesTitles/all), which are groups of similar phenotypes [68]. To this end, we used the binomial test and Bonferroni adjustment on some types of phenotypic series. Fig 8 shows that phenotypes belonging to the same phenotypic series (i.e., “Parkinson disease–PS168600”) are enriched by ten HPO terms including depression (HP:0000716), slow progression (HP:0003677), gait disturbance (HP:0001288), urinary urgency (HP:0000012), psychotic episodes (HP:0000725), hyposmia (HP:0004409), akinesia (HP:0002304), adult-onset (HP:0003581), mask-like facies (HP:0000298), and hyperreflexia (HP:0001347). Most of the enriched phenotype ontology terms were reported to be associated with Parkinson’s disease (PD). Indeed, firstly, PD is a progressive neurodegenerative disease characterized by a decrease in dopamine, resulting in problems of sending motor commands to muscles such as akinesia [69, 70] and hyposmia [71] and mask-like facies [72]. Secondly, depressive disturbances and psychotic episodes are common in patients with PD [73] and [74], respectively. Finally, the disease also relates to urinary dysfunction [75].

## Conclusions and discussion

UFO unifies most of the proposed semantic similarity measures for similarity calculation between ontology terms and between biomedical entities. Besides, it provides enrichment analysis for a set of entities and visualization for terms and annotated entities. These functions can be applied to any type of biomedical ontology. The ability of the tool was demonstrated in various biomedical applications, such as finding the best measures for estimating the similarity between two disease phenotypes based on HPO, building biomedical entity similarity networks for predicting disease-associated biomarkers, and enrichment analysis for a set of similar phenotypes. Comparing with the other tools, UFO provides a comparable number of semantic similarity measures with SML-Toolkit but larger than any others; thus, the user has more choice of selecting semantic measures for their application. Besides, by developing as an app of the Cytoscape platform, UFO can exploit the integration and visualization functions of the platform. The visualization functions of UFO help understand about relationships between ontology terms, annotation, and functional similarity between annotated biomedical entities. Also, UFO can interoperate with other apps and functions of Cytoscape, for which many apps have been developed for various biomedical problems. Moreover, as a GUI tool, UFO does not need the user to have programming ability.

As the ontology data are increasing and the level of annotation is varied among annotated biomedical entities, thus the evaluation of semantic similarity measures should also consider annotation size [76] in the future. In addition, for some measures having high computational complexity such as the hybrid between-term method [41], a parallel implementation should be deployed to improve the analysis time for batch jobs (i.e., estimate thousands to millions of entity pairs using the high computationally complex measures). Finally, UFO should be able to work with other ontology formats (e.g., RDF, OWL) instead of only OBO format.

## Supporting information

S1 Table Ontology tool comparison. (XLSX)

S1 File Semantic similarity measures. (PDF)

S2 File User manual & case studies. (PDF)
