# Supplementary material for: UFO: A tool for unifying biomedical ontology-based semantic similarity calculation, enrichment analysis and visualization
Source: PLoS One. 2020 Jul 9;15(7):e0235670. doi: 10.1371/journal.pone.0235670 (PMC7347127; doi:10.1371/journal.pone.0235670)
Supplement: S1 File — (PDF) [file pone.0235670.s002.pdf]

UFO: a tool for unifying biomedical ontology-based semantic similarity calculation, enrichment analysis and visualization

Duc-Hau Le[1,2,*]

[1]Department of Computational Biomedicine, Vingroup Big Data Institute, Hanoi, Vietnam.

[2]School of Computer Science and Engineering, Thuyloi University, Hanoi, Vietnam.

* To whom correspondence should be addressed. Tel: +84 912 324564; Email: hauldhut@gmail.com

Supplementary File 1

Semantic Similarity Measures

I. Information content 2

II. Between-term measures 3

A. Node-based measures 3

B. Edge-based measures 5

C. Hybrid-based measures 6

III. Between-entity measures 7

A. Pairwise 7

B. Groupwise 8

IV. Enrichment analysis 9

V. Similarity between two entity sets 11

VI. References 12

Biomedical ontologies are represented in a directed acyclic graph (DAG). In this section, we introduce eleven implemented between-term semantic similarity measures (including eight node-based, two edge-based and one hybrid-based) and eleven between-entity measures (including four pairwise-based and seven group-wise-based ones). First, we introduce information content (IC) of a term. Then, we define semantic similarity measures between terms and between entities.

I. INFORMATION CONTENT

The IC of a term is calculated based on a corpus, (*i.e.*, an annotation database of HPO (Köhler, et al., 2014)) as following:

$$IC_t = -\log(p_t)$$

where p_t *is* the probability of a term occurred in a given corpus (Lord, et al., 2003):

$$p_t = \frac{f_t}{t_{root}}$$

where

$$f_t = Annot_t + \sum\nolimits_{c \in Children_t} f_c$$

where $Annot_t$ is number of proteins annotated with term *t* in a corpus and $Children_t$ is the set of children of term *t* in given ontology graph.

Figure 1 illustrates Information Content (IC) calculation for Gene Ontology (GO) term using Gene Ontology and Annotation databases.

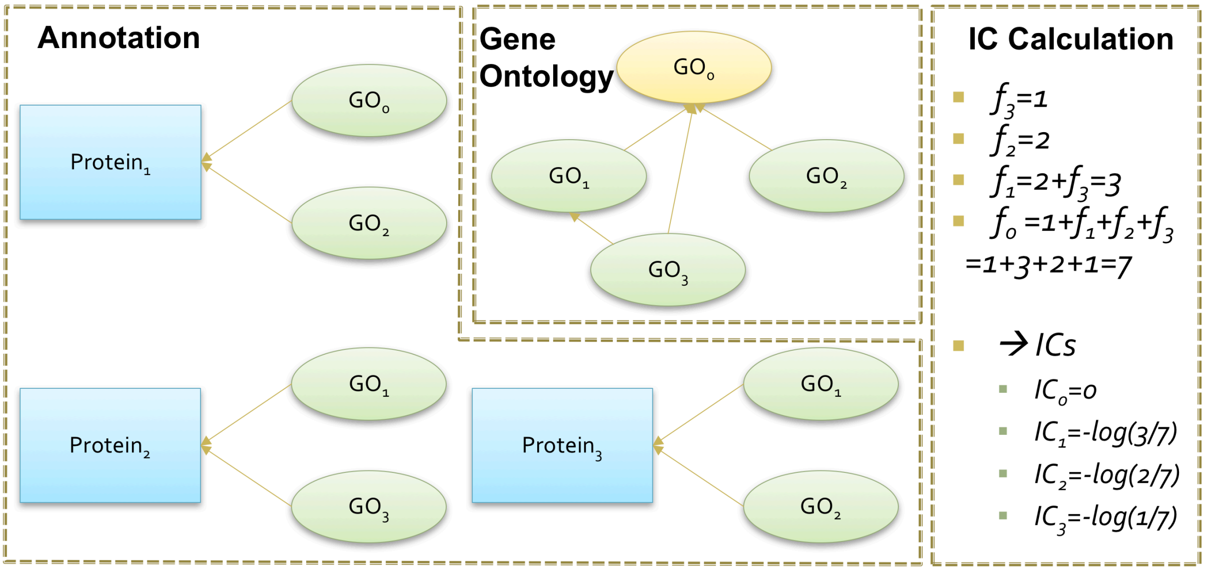

Figure S1: Illustration of Information Content (IC) Calculation for Gene Ontology term using Gene Ontology and Annotation databases.

In the UFO tool, we visualize IC of terms by color. Figure 2 visualizes ICs of some HPO terms by their color (the higher IC of the term is the redder of node is)

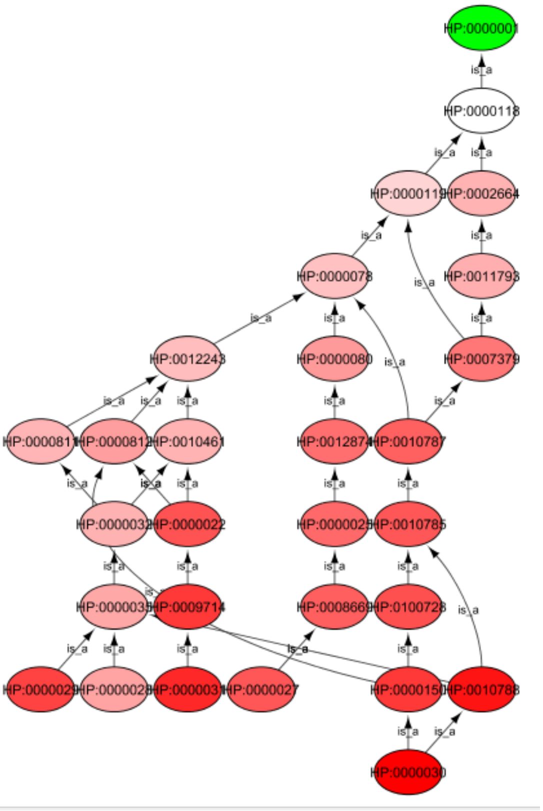

Figure S2: ICs of HPO terms are visualized by red color (blue node is root).

II. Between-term measures

The ontology data is represented in directed acyclic graph, in which each term is located in a node and the relations between terms are represented by edge connecting nodes. Therefore, calculation for semantic similarity between terms are categorized into two main methods: i) node-based and ii) edge-based.

A. Node-based measures

In this section, we firstly introduce four node-based measures for similarity between terms. They are all based on most informative common ancestors (MICA) of the terms, which is defined as following:

$$IC_{MICA} = \max_{c \in SP(t_1,t_2)} (IC_c)$$

where $SP(t_1, t_2)$ are shared ancestors of terms t_1 and t_2.

Figure 3 shows shared ancestors and MICA term of two HPO terms (HP:0000008 and HP:0000010)

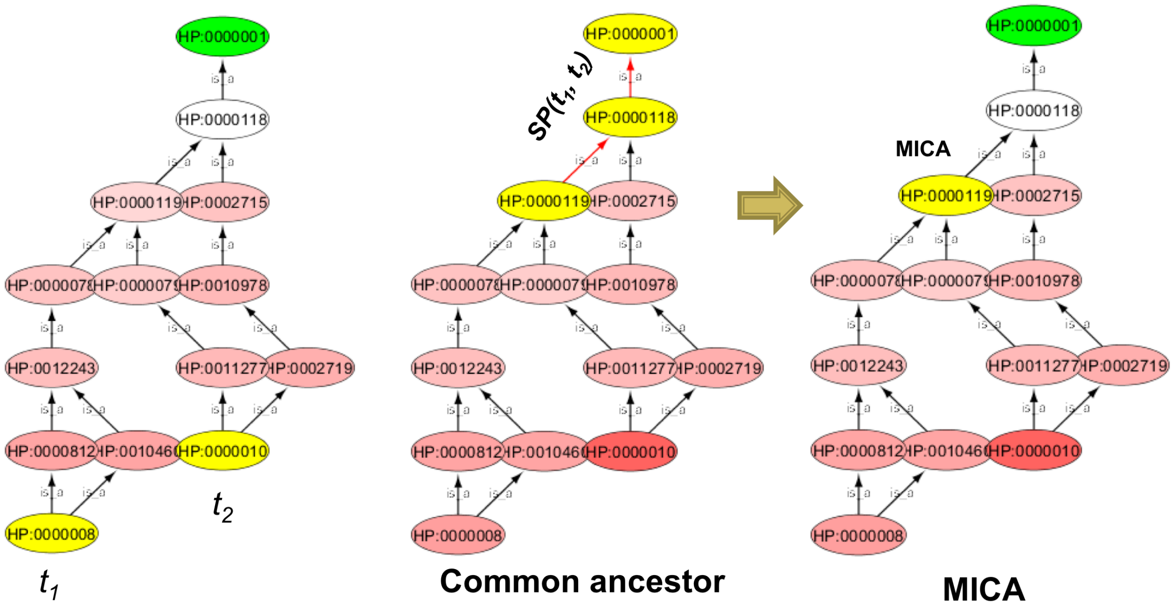

Figure S3: Shared/Common ancestors: Shared ancestors (HP:0000001, HP:0000118 and HP:0000119) and MICA term (HP:0000119) of two HPO terms (HP:0000008 and HP:0000010)

First, Resnik (Resnik, 1995) defined the similarity between two terms as following:

$$IC_t = -\log(p_t)$$

$$SimT_{Resnik}(t_1, t_2) = IC_{MICA}$$

Second, other between-term similarity measures proposed by (Lin, 1998), (Jiang and Conrath, 1997) and (Schlicker, et al., 2006) were defined respectively as following:

$$SimT_{Lin}(t_1, t_2) = \frac{2 \times IC_{MICA}}{IC_{t_1} + IC_{t_2}}$$

$$SimT_{JC}(t_1, t_2) = \frac{1}{IC_{t_1} + IC_{t_2} - 2 \times IC_{MICA} + 1}$$

$$SimT_{Rel}(t_1, t_2) = \frac{2 \times IC_{MICA} \times (1 - P_{MICA})}{IC_{t_1} + IC_{t_2}}$$

where P_{MICA}=$10^{-IC_{MICA}}$

Similarly to the four between-term similarity measures, (Couto, et al., 2005) defined common disjunctive ancestors (CDA) of terms t_1 and t_2 to replace MICA in the four measures as following:

$$IC_{CDA} = \frac{\sum_{t \in CDA} IC_t}{|CDA|}$$

where CDA contain common disjunctive ancestors of terms t_1 and t_2

Figure 4 shows CDA terms (HP:0000118 and HP:0000119) of two HPO terms (HP:0000008 and HP:0000010)

Therefore, four more between-term similarity measures were defined: $SimT_{ResnikGraSM}$, $SimT_{LinGraSM}$, $SimT_{JCGraSM}$ and $SimT_{RelGraSM}$. In summary, a total of eight node-based between-term similarity measures were used in our study.

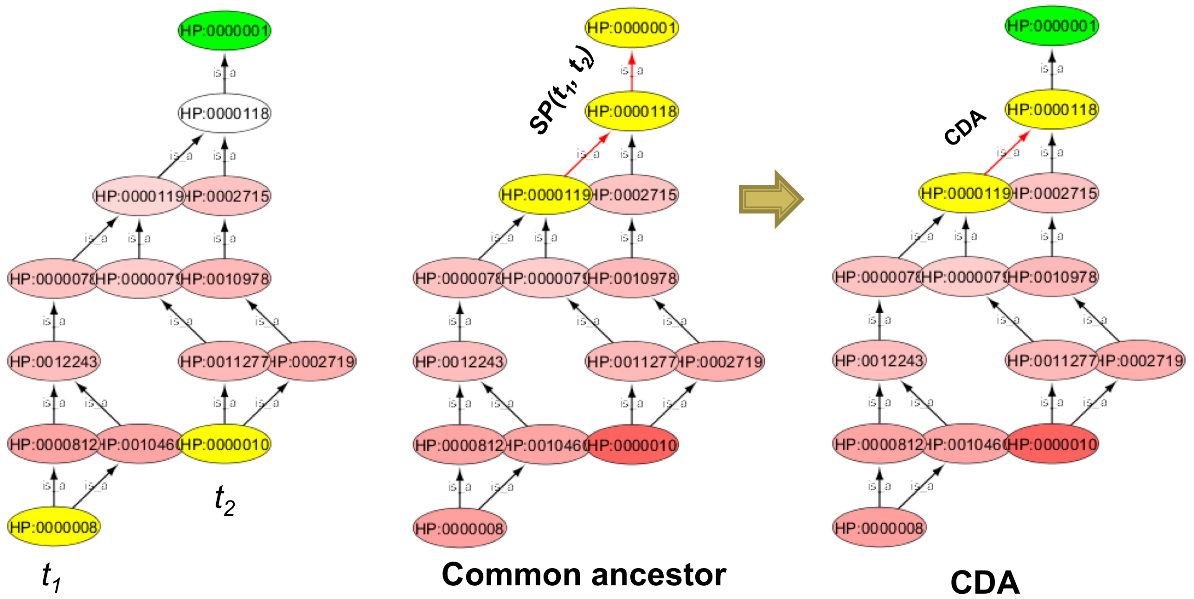

Figure S4: Shared/Common ancestors: Shared ancestors (HP:0000001, HP:0000118 and HP:0000119) and CDA terms (HP:0000118 and HP:0000119) of two HPO terms (HP:0000008 and HP:0000010)

B. *Edge-based measures*

In this section, we introduced two widely used edge-based between-term semantic similarity measures. The first one is simply defined as length of longest path from lowest common ancestor (LCA) to root, $SimT_{Wu}(t_1, t_2) = (LCA, root)$ (Wu, et al., 2005). Meanwhile, the second one (Yu, et al., 2005) additionally takes consider on length of longest path between each term to LCA and formally defined as following:

$$SimT_{Yu}(t_1, t_2) = \frac{L(LCA, root)}{L(LCA, root) + L(t_1, LCA) + L(t_2, LCA)}$$

where $L(t_1, t_2)$ length of the longest path between two terms t_1 and t_2

Figure 5 shows the longest path of two HPO terms (HP:0000008 and HP:0000010).

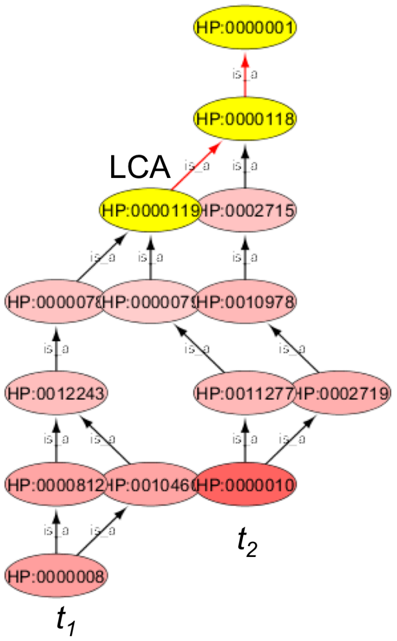

Figure S5: The longest path (red line) of two HPO terms (HP:0000008 and HP:0000010).

C. Hybrid-based measures

We implemented one hybrid measure, which was introduced in (Wang, et al., 2007). In the study, an ontology term t is represented as $DAG_t = (t, T_t, E_t)$ where T_t is the set of ontology terms in DAG_t, including term t and all of its ancestor terms in the ontology graph, and E_t is the set of edges connecting the ontology terms in DAG_t. To quantitatively compare two ontology terms, a semantic value of term t is defined as the aggregate contribution of all terms in DAG_t to the semantics of term t as following:

$$SV(t) = \sum_{t_i \in T_t} S_t(t_i)$$

where

$$S_t(t_i) = \begin{cases} S_t(t) = 1 \\ S_t(t_i) = \max\{w_e * S_t(c) | c \in children_{t_i}\} \;\; if \; t_i \neq t \end{cases}$$

and $0 < w_e < 1$

Finally, a semantic similarity between two terms, t_1 and t_2, is defined as follow:

$$SimT_{Hybrid}(t_1, t_2) = \frac{\sum_{t_i \in T_{t_1} \cap T_{t_2}} (S_{t_1}(t_i) + (S_{t_2}(t_i))}{SV(t_1) + SV(t_2)}$$

Figure 6 shows DAGs of two HPO terms (HP:0000008 and HP:0000010).

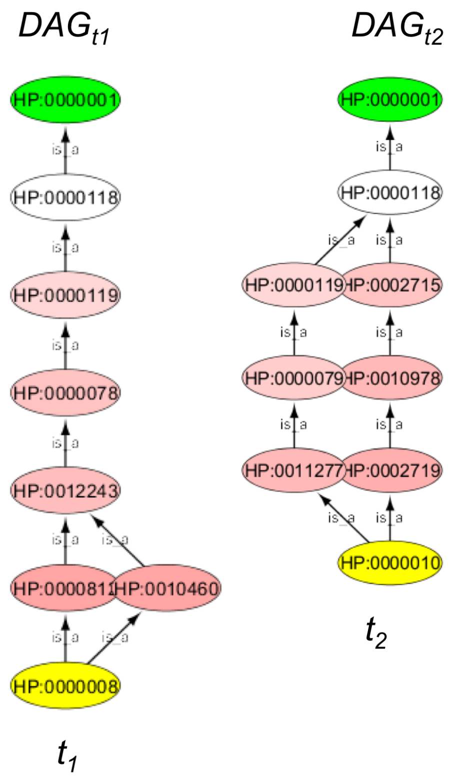

Figure S6: DAGs of two HPO terms (HP:0000008 and HP:0000010).

III. Between-entity measures

For assessment of similarity between two annotated entities, two main approaches have been proposed, i.e., pairwise and groupwise.

Assuming that two entities e_1 and e_2 are annotated by a set of term $T_1 = \bigcup_k^m t_{1k}$ and $T_2 = \bigcup_l^n t_{2l}$. In this section, we are going to introduce between-entity similarity measures

A. *Pairwise*

This approach calculates the similarity between two entities based on the similarity of every pair terms which annotate to the entities.

First, the similarity of every pair of terms is calculated to generate a similarity matrix as following (see Fig. 7):

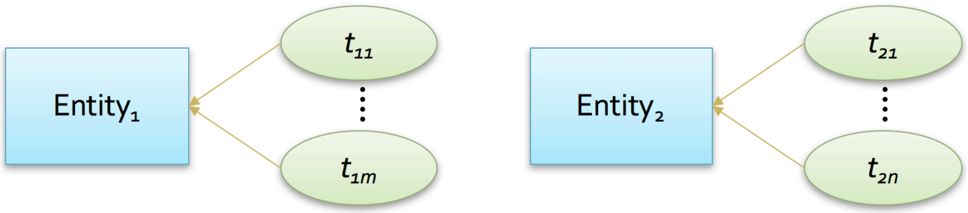

	t_{21}	…	t_{2n}	
t_{11}	$SimT(t_{11}, t_{21})$	…	$SimT(t_{11}, t_{2n})$	$MaxRow_1$
…	…	…	…	…
t_{1m}	$SimT(t_{1m}, t_{21})$	…	$SimT(t_{1m}, t_{2n})$	$MaxRow_m$
	$MaxCol_1$	…	$MaxCol_n$	

Figure S7. Example of a semantic similarity matrix, where shadding cells are elements of the matrix, t_{ij} are column and cell headers, $MaxRow_i$ and $MaxCol_i$ are maximum values of row i and column i, respectively.

Then, four pairwise between-entity were defined as following:

The first two measures were simply either based on average (Lord, et al., 2003) or maximum (Sevilla, et al., 2005) similarity of all pairs.

$$SimE_{Avg} = \underset{i=1\ldots m; j=1\ldots n}{\text{Avg}} (SimT(t_{1i}, t_{2j}))$$

$$Max_{Max} = \underset{i=1\ldots m; j=1\ldots n}{\text{Max}} (SimT(t_{1i}, t_{2j}))$$

Meanwhile, Couto et al (Couto, et al., 2005) and Azuaje et al (Azuaje, et al., 2005) opted for a composite average in which only the best-matching term pairs are considered (best-match average):

$$SimE_{BMA} = \frac{\sum_{i=1\ldots m} MaxRow_i + \sum_{j=1\ldots n} MaxCol_j}{n_{Row} + n_{Col}}$$

Finally, Schlicker et al (Schlicker, et al., 2006) proposed a variation of the best-match average:

$$SimE_{RCMax} = \max\left(\underset{i=0\ldots m}{\text{Avg}} (MaxRow_i), \underset{j=0\ldots n}{\text{Avg}} (MaxCol_j)\right)$$

B. Groupwise

This approach is categorized into two main methods: i) vector-based and ii) graph-based. In which, two popular vector-based between-entity similarity measures Cosine (Huang, et al., 2007) and Kappa (Chabalier, et al., 2007) were defined based on cosine and kappa coefficients. More specifically, T_1 and T_2 were first represented as binary vectors as following:

$$v_1 = (v_{1_1}, \ldots, v_{1_N}),\ v_2 = (v_{2_1}, \ldots, v_{2_N})$$

$$v_{1_k}, v_{2_l} = \begin{cases} 1 & if\ t_k, t_l \in T \\ 0 & otherwise \end{cases}$$

where T is whole set of N terms in the ontology database.

Then, the cosine of the two vectors v_1 and v_2 is calculated:

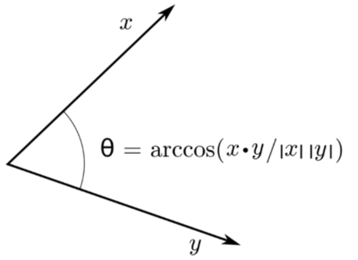

$$SimE_{Cosine}(v_1, v_2) = \frac{\sum_{i=1}^{N}(v_{1_i} \times v_{2_i})}{\sqrt{\sum_{i=1}^{N}(v_{1_i} \times v_{1_i})} \times \sqrt{\sum_{i=1}^{N}(v_{2_i} \times v_{2_i})}}$$

Another vector-based between-entity similarity measure is based on Kappa coefficient. Formally, it is defined as following:

$$Kappa(v_1, v_2) = \frac{P_o - P_e}{1 - P_e}$$

where:

- P_o is observed proportionate agreement: $(P_{11} + P_{00})/P_t$
- P_e is overall probability of random agreement: $P_{e1}+P_{e0}$
- P_{e1} is probability that both of them=1: $P_{v1=1} * P_{v2=1}$
- $P_{v_1=1}$ is probability v_1=1: $(P_{11}+P_{10})/P_t$
- $P_{v_2=1}$ is probability v_2=1: $(P_{11}+P_{01})/P_t$
- P_{e0} is probability that both of them = 0: $P_{v1=0} * P_{v2=0}$
- $P_{v_1=0}$ is probability v_1=0: $(P_{01}+P_{00})/P_t$
- $P_{v_2=0}$ is probability v_2=0: $(P_{10}+P_{00})/P_t$
- P_t, P_{11}, P_{00}, P_{10} and P_{01} is total number of observations, number of observations where $v_{1_i} = v_{2_i} = 1$, number of observations where $v_{1_i} = v_{2_i} = 0$, number of observations where $v_{1_i} = 1$ and $v_{2_i} = 0$, and number of observations where $v_{1_i} = 0$ and $v_{2_i} = 1$, respectively.

		v_2		
		1	0	
v_1	1	P_{11}	P_{10}	
	0	p_{01}	p_{00}	
				P_t

For graph-based measures, T1 and T2 is extended with ancestors of terms in each set. Therefore, they are defined as follows:

$$T_1 = \bigcup_{k}^{m} \left(t_k \cup anc(t_k)\right)$$

$$T_2 = \bigcup_{l}^{n} \left(t_l \cup anc(t_l)\right)$$

Five more graph-based between-entity similarity measures were defined as following:
A measure is based on term overlap (Lee, et al., 2004)

$$SimE_{TO} = |T_1 \cap T_2|$$

A normalized version of $SimE_{TO}$ (Mistry and Pavlidis, 2008)

$$SimE_{NTO} = \frac{|T_1 \cap T_2|}{min(|T_1|, |T_2|)}$$

A measure is based on Jaccard index (Gentleman, 2005; Martin, et al., 2004)

$$SimE_{UI} = \frac{|T_1 \cap T_2|}{|T_1 \cup T_2|}$$

An IC-based weighted version of $SimE_{UI}$ (Pesquita, et al., 2007)

$$SimE_{GIC} = \frac{\sum_{t_k \in |T_1 \cap T_2|} IC_{t_k}}{\sum_{t_l \in |T_1 \cup T_2|} IC_{t_l}}$$

Finally, a longest path-based between-entity similarity measure (Gentleman, 2005):

$$SimE_{LP} = \max_{t_k \in |T_1 \cap T_2|} L(t_k, root)$$

In summary, a total of eleven between-entity similarity measures were used in our study.

IV. Enrichment analysis

Given an entity set (S_e) and an ontology term t, let H_0 denotes the null hypothesis that there is no significant association between S_e and t. The association between S_e and t is defined as an overlap (k) between the S_e and the set of entities annotated with term t in the corpus. There are

three statistical tests popularly used to test whether the overlap is significant or not (i.e., H_0 is rejected or not) (Rivals, et al., 2006), i.e., Fisher's exact test (equivalent to Hypergeometric test), Binomial test and Chi-squared test. In UFO, we implemented the Fisher's exact test and the Binomial test.

The above problem can be formulated as following 2×2 contingency table.

	Entity set (S_e)	Non-entity set (the remaining)	
Entities annotated with *t*	*a* (*k*)	*b*	*a*+*b* (*K*)
Entities not annotated with *t*	*c* (*n*-*k*)	*d*	*c*+*d* (*N*-*K*)
	a+*c* (*n*)	*b*+*d*	*N*

Fisher showed that the probability of obtaining the overlap (with an observed value k) was given by the hypergeometric distribution with parameters *N*, *n*, and *K* (Agresti, 1992; Fisher, 1922):

$$P_{(X=k)} = \frac{\binom{a+b}{a}\binom{c+d}{c}}{\binom{N}{a+c}} = \frac{\binom{K}{k}\binom{N-K}{n-k}}{\binom{N}{n}}$$

where:

- *N* is number of annotated entities in the corpus (e.g., number of genes in the corpus which are annotated with GO terms)
- *K* is number of entities annotated with term *t* in the corpus (e.g., number of genes in the corpus which are annotated by a specific GO term).
- *n* is number of entities in the entity set (S_e) (e.g., a gene set of interest which we want to find GO terms significantly annotating to).
- *k* is number of entities in the entity set which are annotated with term *t*.

For a large sample, the overlap has approximately a binomial distribution

$$P_{(X=k)} = \binom{K}{k} p^k (1-p)^{n-k}$$

where:

- p is success probability in the population (i.e., *K*/*N*, the probability that an entity is annotated with term *t* in the corpus).
- *K* is the number of success states in the population (i.e., number of entities annotated with term *t* in the corpus).
- *n* is the number of draws (i.e., number of entities in the entity set (S_e)).
- *k* is the number of observed successes (i.e., number of entities in the entity set which are annotated with term *t*).

S_e is said to be enriched by *t* if there is statistically significant overlap between entity set (S_e) (*n*) and the set of entities annotated with term *t* in the corpus (*K*).

When testing multiple hypotheses, the obtained p-values have to be corrected in order to control the type I error (false positive) rate (Noble, 2009). In UFO, we implemented two multiple testing correction methods, i.e., Bonferroni, and Benjamini and Hochberg correction. The former (i.e., Bonferroni) strongly controls the probability of making at least one type I error (i.e., the family-wise error rate (FWER)) for tests (Bonferroni, et al., 1936); meanwhile, the latter is to control the false discovery rate (FDR), i.e. the expected proportion of false positives among the positively identified tests (Benjamini YaY, 2001).

After applying a multiple testing correction method, an adjusted p-value was obtained for each ontology term *t*. The p-value represents the probability of the null hypothesis; thus, the smaller p-value is the less likely that the association between the entity set (S_e) and that term is random. In enrichment analysis, the p-value ≤0.05 indicates the association is statistically significant.

V. Similarity between two entity sets

The procedure to calculate the similarity between two sets of entities is as following:

- For each set of entities, a set of ontology terms statistically significant annotating for the entity set is identified. Thus, each entity set is now equivalent to a meta-entity which is annotated with the set of significant ontology terms.
- The similarity between two entity sets is now equivalent to the similarity between two meta-entities, that can be calculated by any of between-entity similarity measures (see section III. BETWEEN-ENTITY MEASURES).

VI. References

Agresti, A. A Survey of Exact Inference for Contingency Tables. *Statist. Sci.* 1992;7(1):131-153.

Azuaje, F., Wang, H. and Bodenreider, O. Ontology-driven similarity approaches to supporting gene func-tional assessment. *Proc. Of The Eighth Annual Bio-Ontologies Meeting* 2005;Michigan, 25 June.

Benjamini YaY, D. the control of false discovery rate in multiple testing under dependency. *Ann Stat* 2001;29.

Bonferroni, C.E., Bonferroni, C. and Bonferroni, C. Teoria statistica delle classi e calcolo delle probabilita'. 1936.

Chabalier, J., Mosser, J. and Burgun, A. A transversal approach to predict gene product networks from ontology-based similarity. *BMC Bioinformatics* 2007;8(1):235.

Couto, F., Silva, M.r. and Coutinho, P. Semantic similarity over the gene ontology: family correlation and selecting disjunctive ancestors. In, *CIKM '05: Proceedings of the 14th ACM international conference on Information and knowledge management*. Bremen, Germany: ACM; 2005. p. 343-344.

Fisher, R.A. On the Interpretation of χ² from Contingency Tables, and the Calculation of P. *Journal of the Royal Statistical Society* 1922;85(1):87-94.

Gentleman, R. Visualizing and distances using GO. *URL* http://www/. *bioconductor. org/docs/vignettes. html* 2005;38.

Huang, D., *et al.* The DAVID Gene Functional Classification Tool: a novel biological module-centric algorithm to functionally analyze large gene lists. *Genome Biology* 2007;8(9):R183.

Jiang, J.J. and Conrath, D.W. Semantic Similarity Based on Corpus Statistics and Lexical Taxonomy. In, *International Conference Research on Computational Linguistics (ROCLING X)*. 1997. p. 9008.

Köhler, S., *et al.* The Human Phenotype Ontology project: linking molecular biology and disease through phenotype data. *Nucleic acids research* 2014;42(D1):D966-D974.

Lee, H., *et al.* Coexpression Analysis of Human Genes Across Many Microarray Data Sets. *Genome Research* 2004;14(6):1085-1094.

Lin, D. An Information-Theoretic Definition of Similarity. In, *Proceedings of the Fifteenth International Conference on Machine Learning*. Morgan Kaufmann Publishers Inc.; 1998.

Lord, P.W., *et al.* Investigating semantic similarity measures across the Gene Ontology: the relationship between sequence and annotation. *Bioinformatics* 2003;19(10):1275-1283.

Martin, D., *et al.* GOToolBox: functional analysis of gene datasets based on Gene Ontology. *Genome Biology* 2004;5(12):R101.

Mistry, M. and Pavlidis, P. Gene Ontology term overlap as a measure of gene functional similarity. *BMC Bioinformatics* 2008;9(1):327.

Noble, W.S. How does multiple testing correction work? *Nature Biotechnology* 2009;27:1135.

Pesquita, C., *et al.* Evaluating GO-based semantic similarity measures. In, *Proc. 10th Annual Bio-Ontologies Meeting*. 2007. p. 38.

Resnik, P. Using information content to evaluate semantic similarity in a taxonomy. In, *Proceedings of the 14th international joint conference on Artificial intelligence - Volume 1*. Montreal, Quebec, Canada: Morgan Kaufmann Publishers Inc.; 1995.

Rivals, I., *et al.* Enrichment or depletion of a GO category within a class of genes: which test? *Bioinformatics* 2006;23(4):401-407.

Schlicker, A., *et al.* A new measure for functional similarity of gene products based on Gene Ontology. *BMC Bioinformatics* 2006;7(1):302.

Sevilla, J.L., *et al.* Correlation between gene expression and GO semantic similarity. *Computational Biology and Bioinformatics, IEEE/ACM Transactions on* 2005;2(4):330-338.

Wang, J.Z., *et al.* A new method to measure the semantic similarity of GO terms. *Bioinformatics* 2007;23(10):1274-1281.

Wu, H., *et al.* Prediction of functional modules based on comparative genome analysis and Gene Ontology application. *Nucleic acids research* 2005;33(9):2822-2837.

Yu, H., *et al.* Broadly predicting specific gene functions with expression similarity and taxonomy similarity. *Gene* 2005;352(0):75-81.
