# Supplementary material for: UFO: A tool for unifying biomedical ontology-based semantic similarity calculation, enrichment analysis and visualization
Source: PLoS One. 2020 Jul 9;15(7):e0235670. doi: 10.1371/journal.pone.0235670 (PMC7347127; doi:10.1371/journal.pone.0235670)
Supplement: S2 File — (PDF) [file pone.0235670.s003.pdf]

UFO: a tool for unifying biomedical ontology-based semantic similarity calculation, enrichment analysis and visualization

Duc-Hau Le[1,2,*]

[1]Department of Computational Biomedicine, Vingroup Big Data Institute, Hanoi, Vietnam.

[2]School of Computer Science and Engineering, Thuyloi University, Hanoi, Vietnam.

* To whom correspondence should be addressed. Tel: +84 912 324564; Email: hauldhut@gmail.com

Supplementary File 2

User Manual & Case Studies

(UFO was tested to be worked well on both Windows, Mac and Ubuntu operating systems)
The following demonstration was performed on Windows 10

1 Setting up **2**
2 How to use **4**
2.1 Prepare data **4**
2.2 Menu Items **4**
2.3 Load and Prepare data **6**
2.4 Main functions **10**
2.4.1 Term-related Functions 10
a. Calculate Semantic Similarity Matrix 10
b. Visualize terms and annotated entities 12
2.4.2 Entity-related Functions 15
a. Calculate Functional Similarity Matrix 16
c. Analyze term enrichment 18
d. Visualize terms and annotated entities 20
e. Assess functional similarity between entity sets 24
2.5 Utilities **25**
2.5.1 Extract annotation data 25
a. Gene Ontology (GO) To Gene 25
b. Human Phenotype Ontology (HPO) To Phenotype 26
c. Disease Ontology (DO) To Gene 26
2.5.2 Weigh entity network 27
3 CASE STUDY **31**
3.1 Assessing human disease phenotype similarity based on ontology **31**
3.2 Construct gene and protein complex similarity network using GO for predicting disease-associated genes and protein complexes **32**
3.3 Construct disease similarity network using HPO and DO for predicting disease-associated genes and lncRNAs **33**
4 Reference **36**

1 SETTING UP

- Install Cytoscape version 3.x (3.6.0 or later).
- Open **App Manager…** in **Apps** menu
- Click on button **Install from File…**
- Choose file **UFO.jar** to install app in Cytoscape
    - **UFO.jar** can be downloaded at https://sourceforge.net/projects/ufo-cytoscape/files/
- The app will be displayed in **Currently Installed** tab

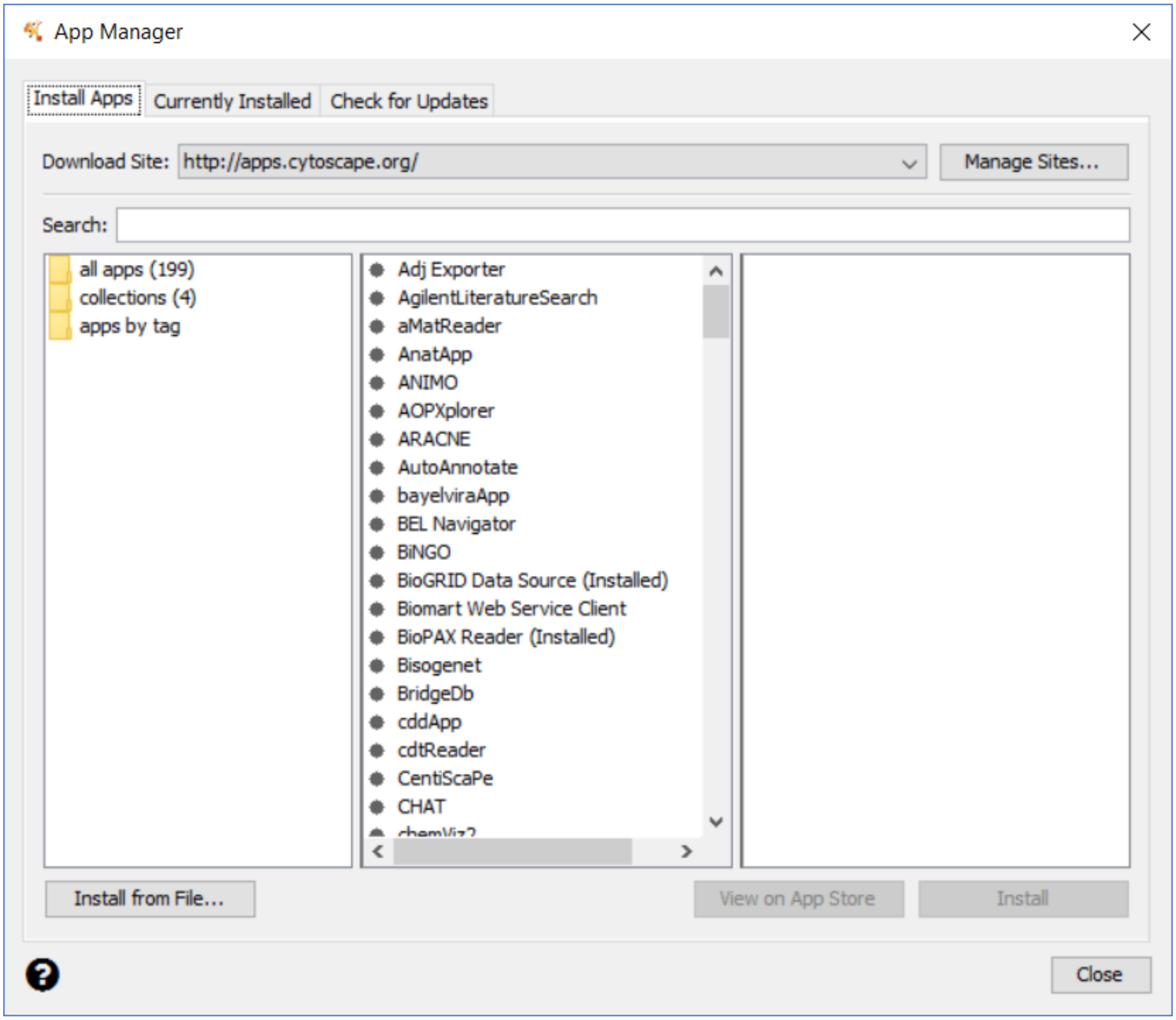

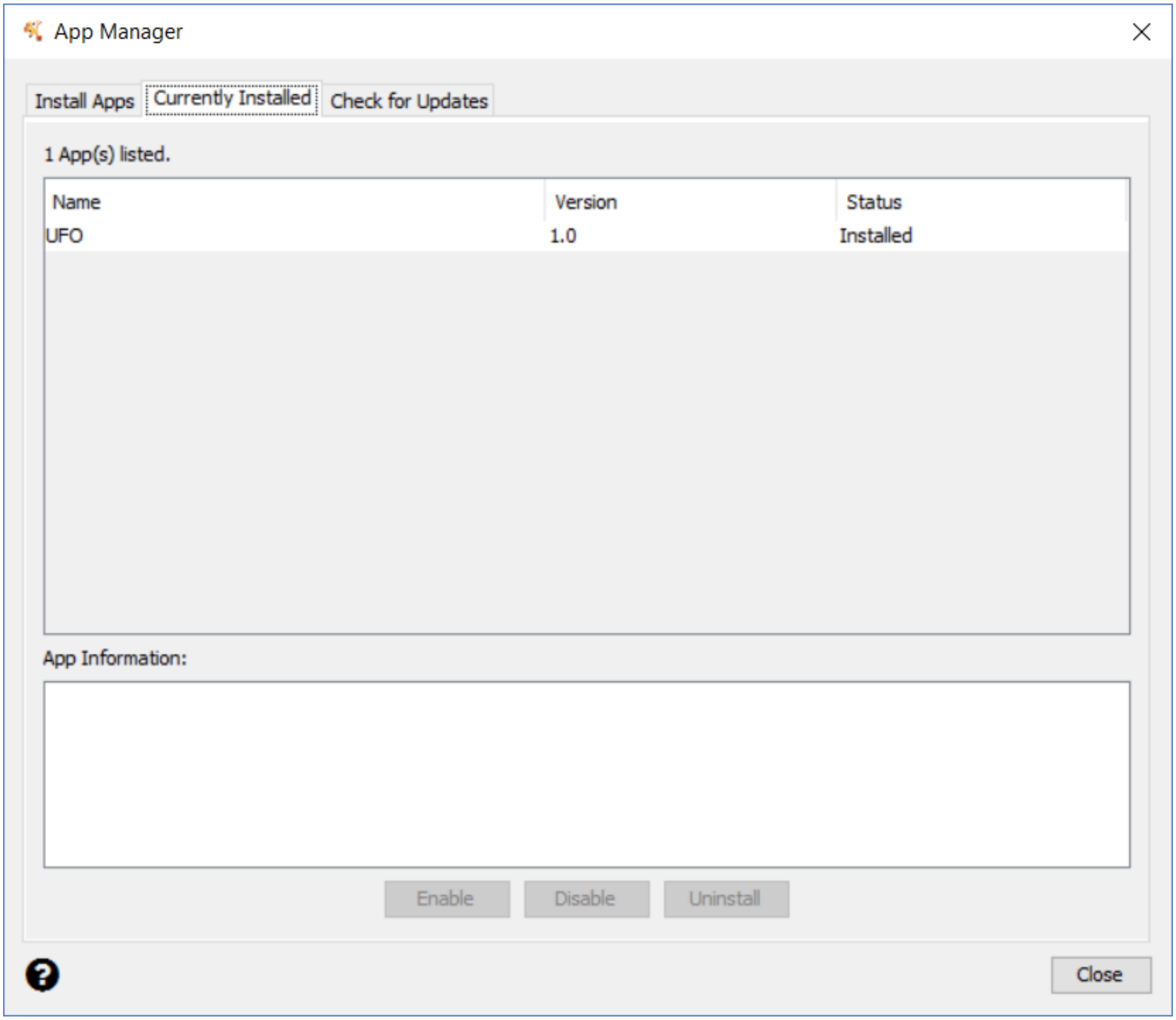
App Manager
Install Apps
Currently Installed
Check for Updates
1 App(s) listed.
Name
Version
Status
UFO
1.0
Installed
App Information:
Enable
Disable
Uninstall
Close

2 HOW TO USE

2.1 Prepare data

- Create a **Data** folder in Cytoscape folder (e.g., "C:\Program Files\Cytoscape_v3.5.1" in Windows, "Applications\Cytoscape_v3.5.1" in Mac)
- Download ontology and annotation data from public resources to **Data** folder
  - All the data can be downloaded at https://github.com/hauldhut/ufo
    - GO (go.obo), HPO (hp.obo) and DO (HumanDO.obo) in **Ontology** folder
    - Original annotation data in **Annotation** folder
    - Preprocessed annotation data in **Annotation-Preprocessed** folder

      → ***Copy all ontology and preprocessed annotation data to Data folder***

  - Otherwise, user can download the all data from original resources
    - For Ontology data.
      - Download Gene Ontology (go.obo) at http://www.geneontology.org/
      - Human Phenotype Ontology (hp.obo), Human Disease Ontology (doid.obo) and other ontologies at The Open Biological and Biomedical Ontologies (http://www.obofoundry.org/)
    - For Annotation data
      - User can download Gene Ontology to Gene (gene2go) at NCBI FTP site (ftp.ncbi.nlm.nih.gov/gene/DATA/gene2go.gz), Disease Ontology to Gene (IDMappings.rdf) at http://dga.nubic.northwestern.edu/ and Human Phenotype Ontology to Phenotype (phenotype_annotation_hpoteam.tab) at http://www.human-phenotype-ontology.org/.
      - Construct other annotation data upon on your needs as following format
        - EntityID<tab>OntologyTermID<tab>EvidenceCode (optional)
          - Where Entity can be Gene, Protein, Phenotype, etc...
          - Ontology can be GO, DO, HPO, etc...
          - EvidenceCode is evidence code of that annotation. Leave blank when no evidence can be specified

2.2 Menu Items

- Run Cytoscape, and the **UFO** application will be automatically loaded in the **Apps** menu

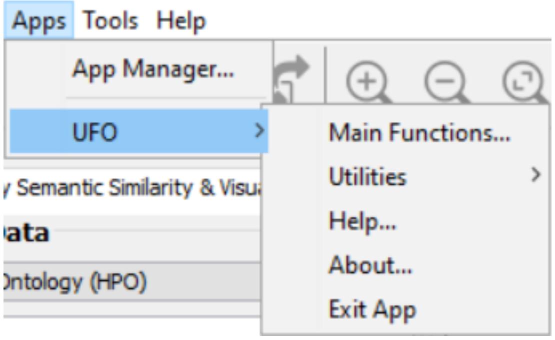

- Menu Items
  - Main Functions…
    - Main functions of this app include calculation of between-term, between-entity similarity matrix; visualization of ontology and annotation data; analysis of term enrichment of a set of entities (e.g., genes); comparison of two entity sets; construction of functional similarity entity network.
  - Utilities

        - Contains a set of utilities which facilitates users to pre-process some data
    - Help...
        - Link to user manual
    - About...
        - Brief information of this app
    - Exit Application
        - To exit this app from Cytoscape panel

2.3 Load and Prepare data

- Click on **Main Functions…** menu item

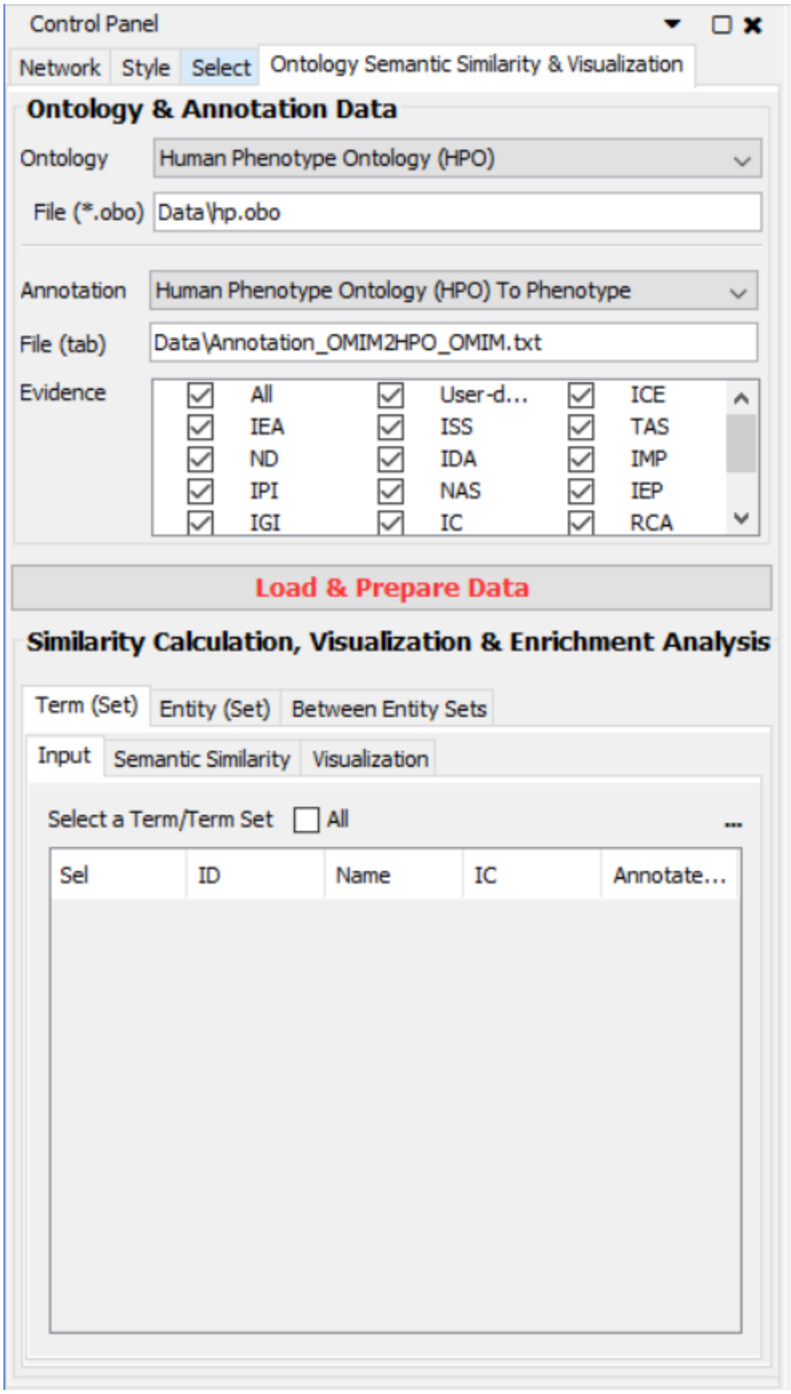

- Select Ontology data from Ontology

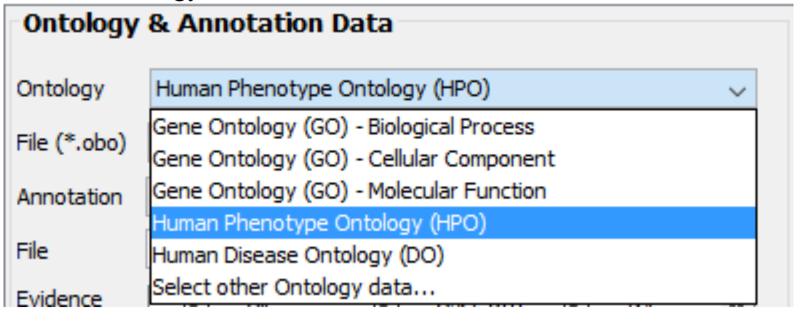

  - We listed some popular Biomedical Ontologies such as Gene Ontology, Human Disease Ontology and Human Phenotype Ontology. Users should download them from public resources as shown in **2.1 Prepare data** section.
  - For other ontology data, user should select **Select other Ontology data...**

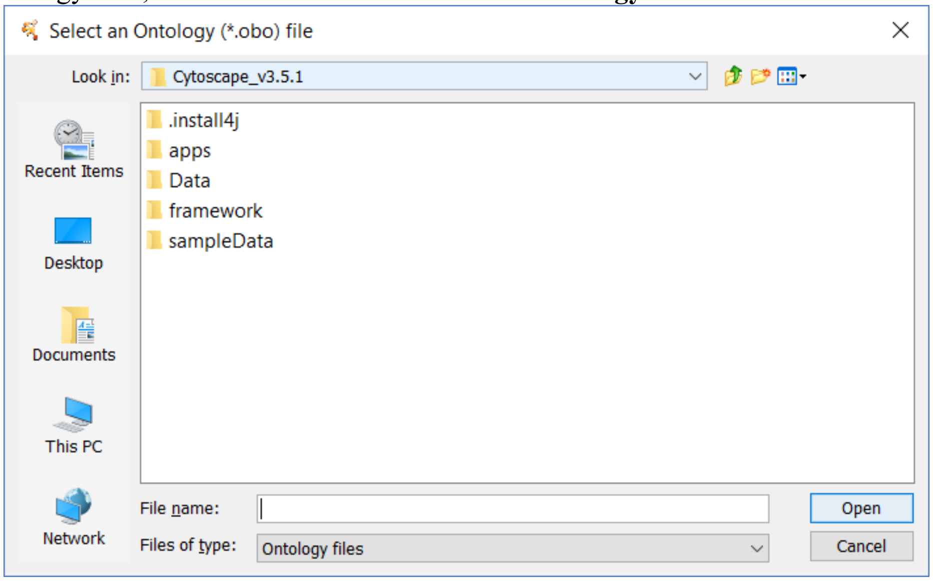

- Select relevant Annotation data from

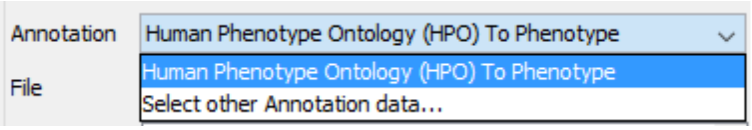

  - We also listed some Annotation data such as Gene Ontology to Genes, Disease Ontology to Genes and Human Phenotype Ontology to Phenotypes for ontologies such as Gene Ontology, Human Disease Ontology and Human Phenotype Ontology, respectively.
  - For other ontology data, user should select **Select other Annotation data...**

- Select Evidence codes

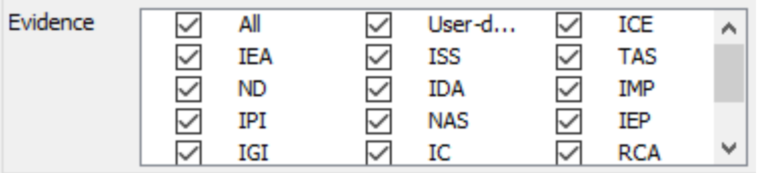

  - Select **All** for all annotations from selected Annotation data, otherwise select individual Evidence code. In addition, user can define their own Evidence codes accordingly by modifying **User-defined** cell or any other cells if they are not presented in the list.

- Load and Prepare Data for Analyses by clicking on

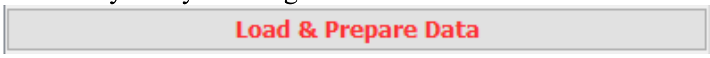

  - This will load Ontology and Annotation data
  - Calculate Information Content (IC) for each Ontology term
  - Term ID, Term Name, IC and annotated Entities will be displayed in

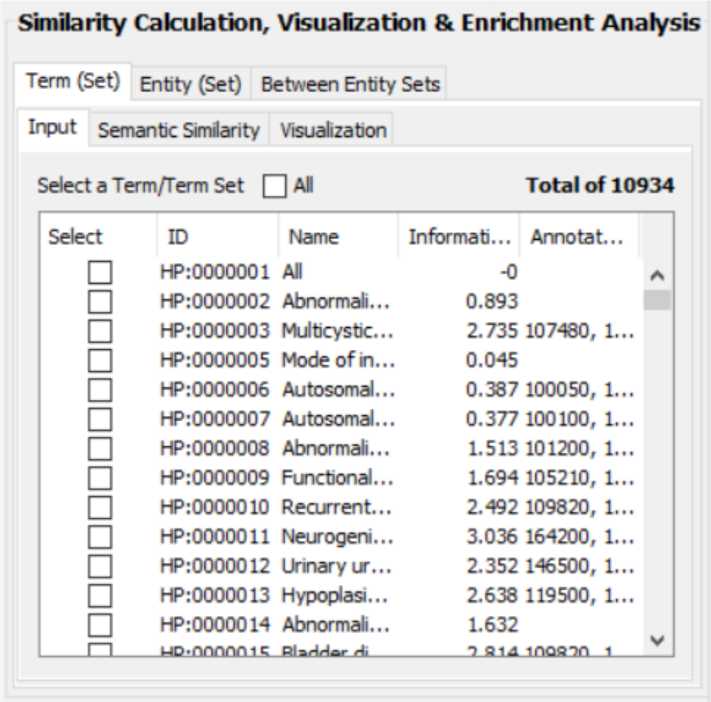

  - Entity ID, Entity Name and annotating Terms will be displayed in

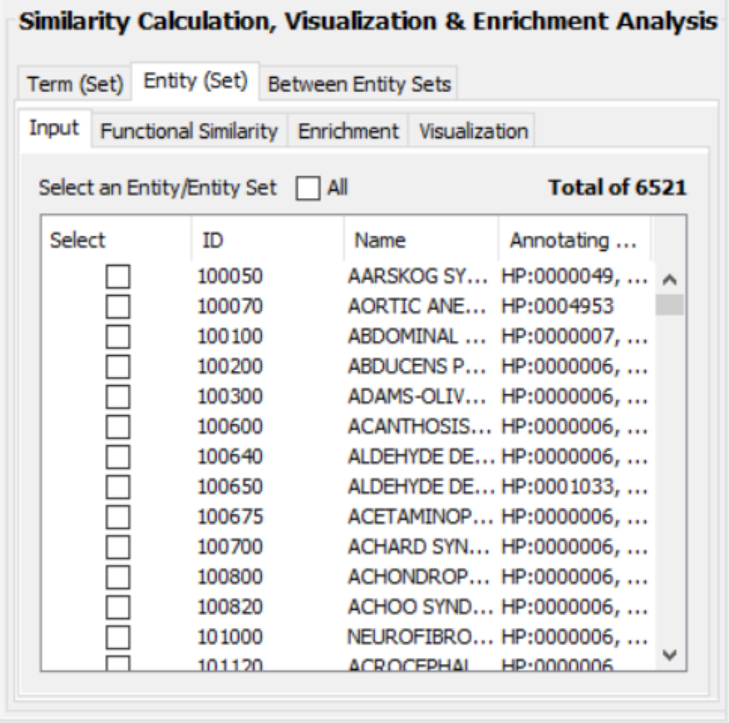

These two lists of Terms and Entities will be used to select terms and entities of interest for further analyses

2.4 Main functions

2.4.1 Term-related Functions

This includes *Semantic Similarity Calculation* and *Visualization*

a. Calculate Semantic Similarity Matrix

<u>Step 1:</u> Select **Term (Set)** tab, then select a set of terms of interest in **Input** tab

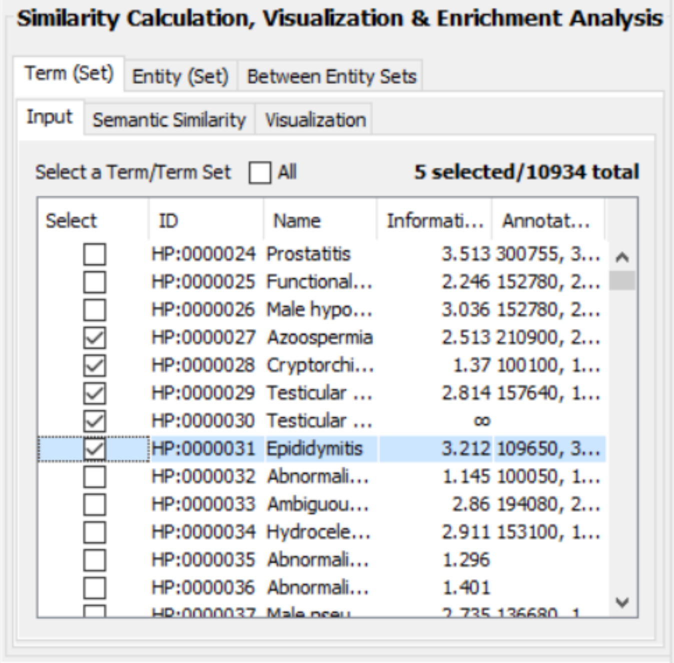

- Note that
  - For *Semantic Similarity Calculation*: Select at least two terms.

<u>Step 2:</u> Select **Semantic Similarity** tab to calculate semantic similarity matrix of the selected terms

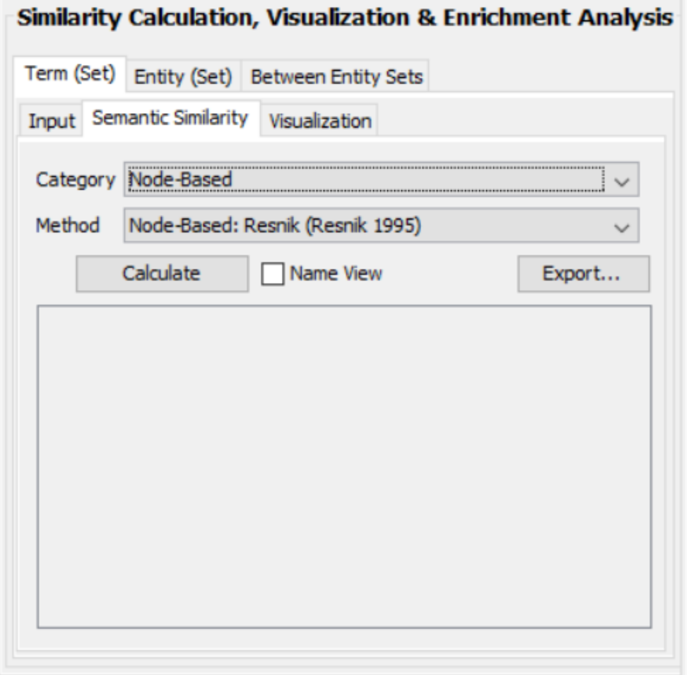

- Select **Category** of methods to calculate semantic similarity between two terms (Pesquita, et al., 2009)

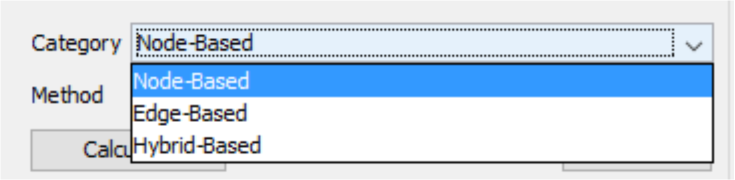

- Select **Method** in the selected **Category**

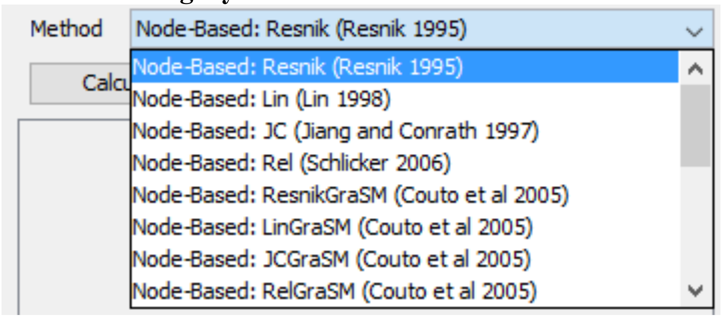

- Click Calculate to calculate semantic similarity matrix

➔ Result (e.g., for *Node-based: Lin (Lin 1998)* method)

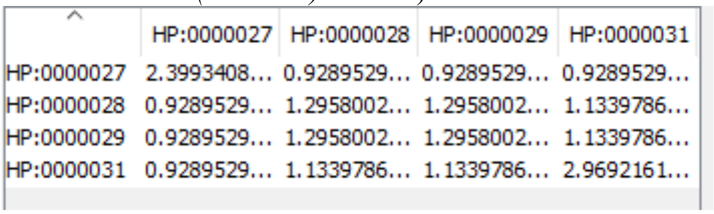

	HP:0000027	HP:0000028	HP:0000029	HP:0000031
HP:0000027	2.3993408…	0.9289529…	0.9289529…	0.9289529…
HP:0000028	0.9289529…	1.2958002…	1.2958002…	1.1339786…
HP:0000029	0.9289529…	1.2958002…	1.2958002…	1.1339786…
HP:0000031	0.9289529…	1.1339786…	1.1339786…	2.9692161…

Check ☐ Name View to turn to Term Name view of the semantic similarity matrix

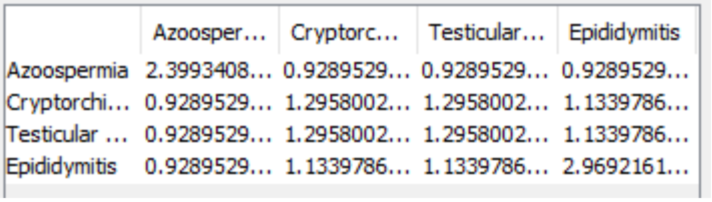

	Azoosper…	Cryptorc…	Testicular…	Epididymitis
Azoospermia	2.3993408…	0.9289529…	0.9289529…	0.9289529…
Cryptorchi…	0.9289529…	1.2958002…	1.2958002…	1.1339786…
Testicular …	0.9289529…	1.2958002…	1.2958002…	1.1339786…
Epididymitis	0.9289529…	1.1339786…	1.1339786…	2.9692161…

- Click Export… to export to tab delimited-text file (e.g., SemMat.txt)

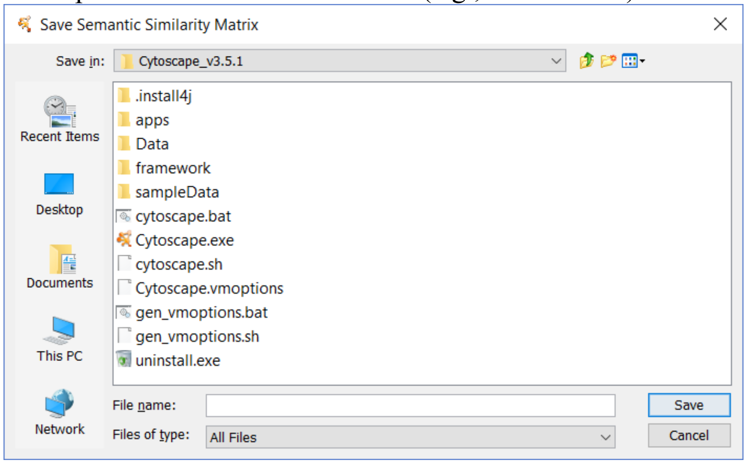

Detail of SemMat.txt file

```
ID	HP:0000027	HP:0000028	HP:0000029	HP:0000031
HP:0000027	2.3993408522366417	0.9289529801759476	0.9289529801759476	0.9289529801759476
HP:0000028	0.9289529801759476	1.2958002603295722	1.2958002603295722	1.1339786867928963
HP:0000029	0.9289529801759476	1.2958002603295722	1.2958002603295722	1.1339786867928963
HP:0000031	0.9289529801759476	1.1339786867928963	1.1339786867928963	2.9692161601932026
```

b. Visualize terms and annotated entities

Step 1: Select **Term (Set)** tab, then select a set of Terms of interest in **Input** tab

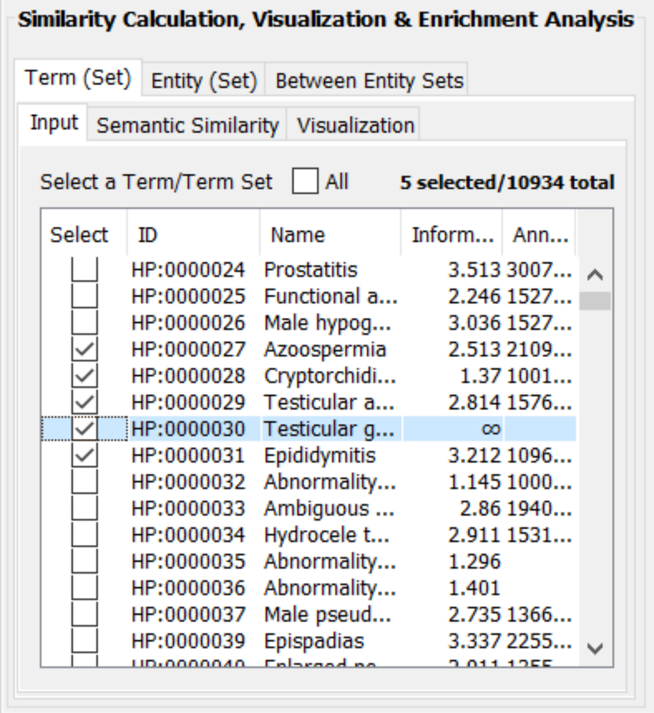

Step 2: Select **Visualization** tab to visualize terms and annotated entities

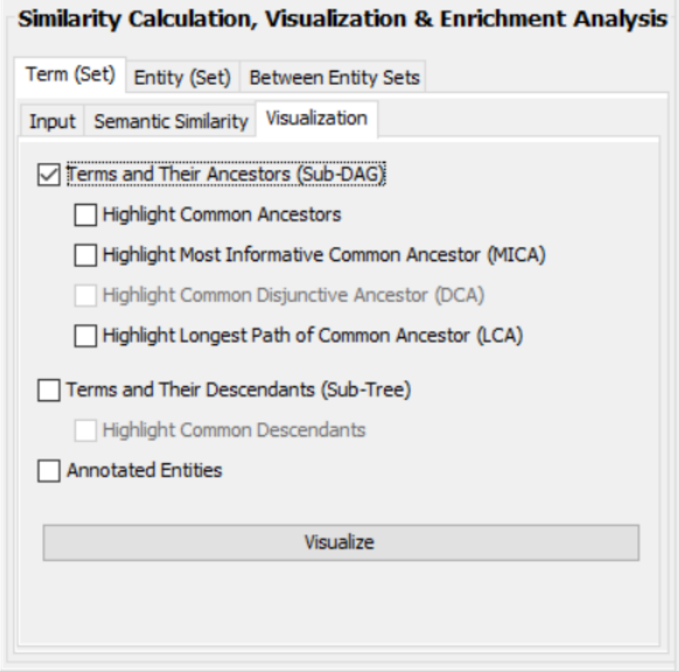

- Select options then click 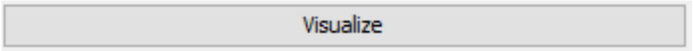

➔ Result

- With **Terms and Their Ancestors**

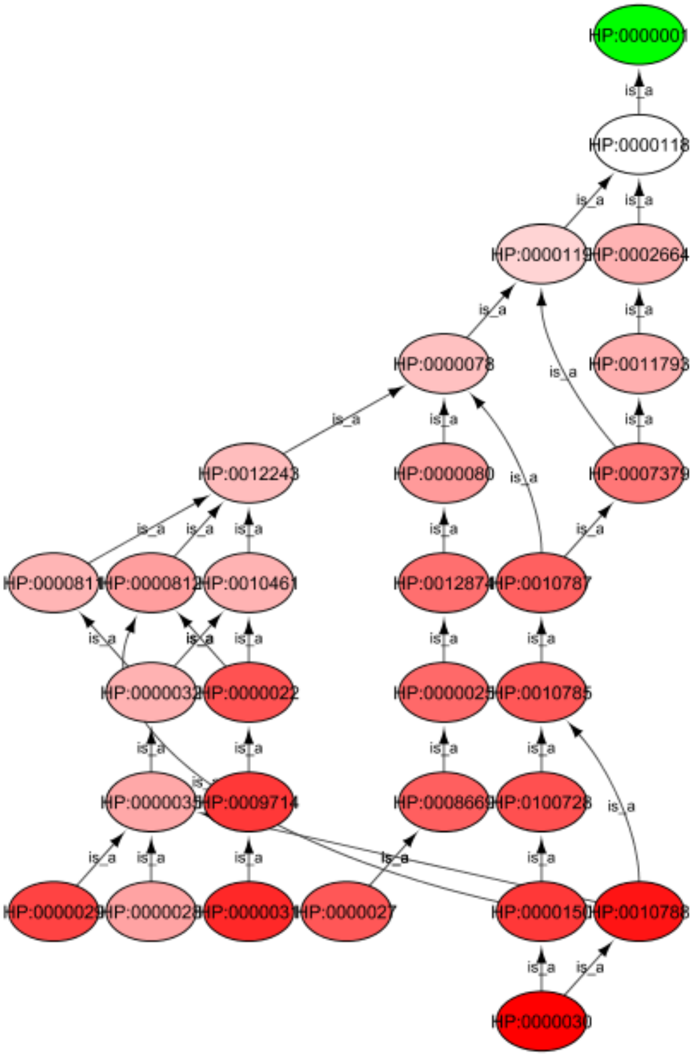

Five terms HP:0000027, HP:0000028, HP:0000029, HP:0000030 and HP:0000031 and their ancestors

- Check on 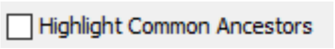

to highlight Common Ancestor terms of the selected terms.

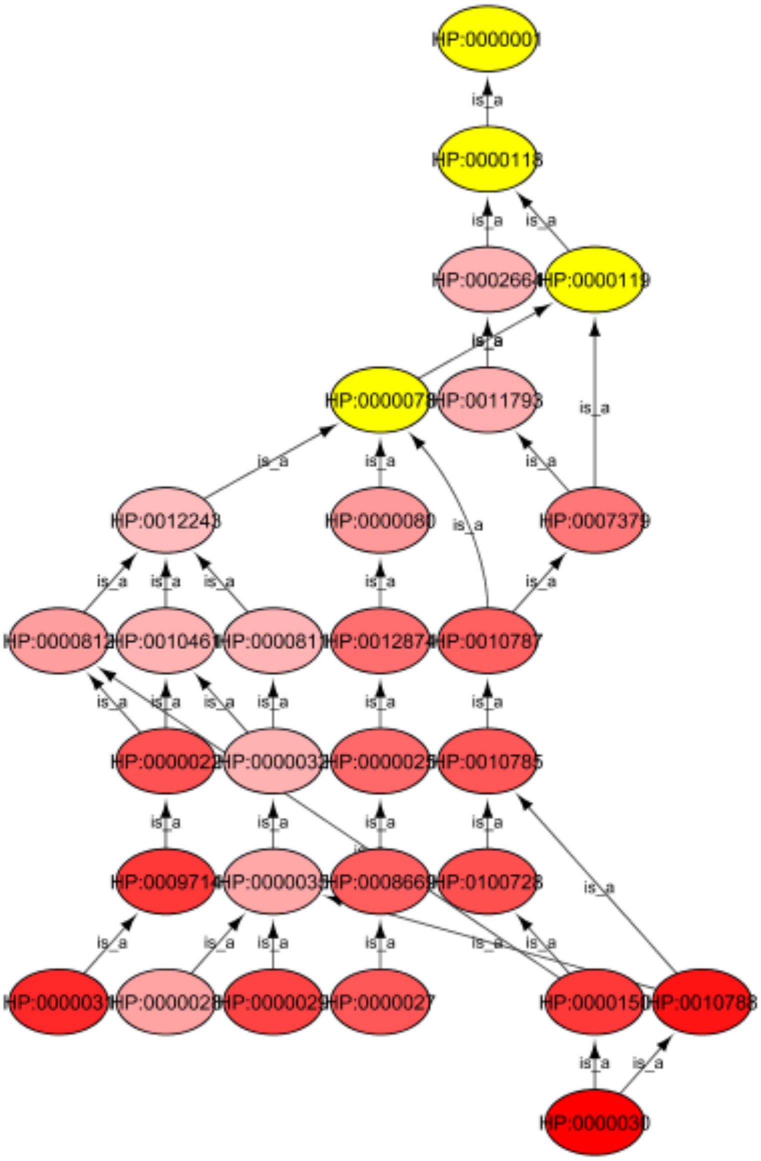

Common ancestors of five terms HP:0000027, HP:0000028, HP:0000029, HP:0000030 and HP:0000031

- With **Terms and Their Descendants**

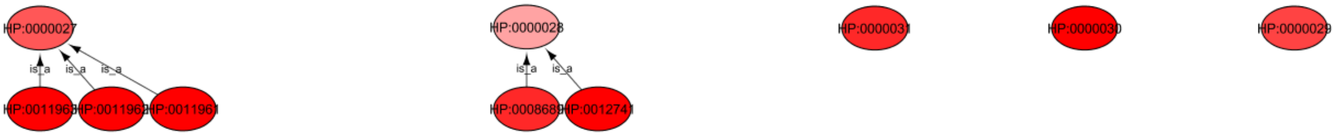

Five terms HP:0000027, HP:0000028, HP:0000029, HP:0000030 and HP:0000031 and their descendants

- Check on 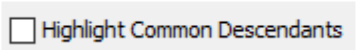

to highlight Common Descendant terms of the selected terms.

- With **Annotated Entities** (e.g., with Term HP: 0000027). User can view all annotated entities of the selected terms from Annotation data.

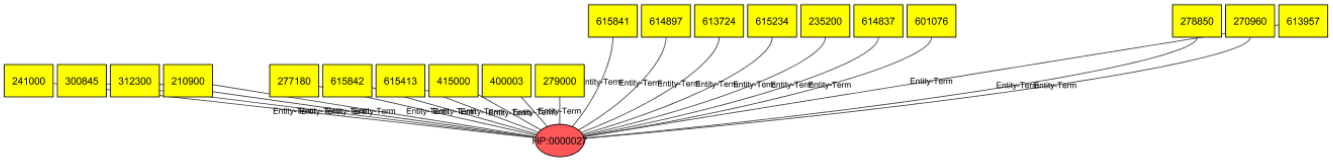

- Note: We provided both ID and Name for each entity/term as their attributes in **Node Table** tab of Cytoscape

ID	Name
**HP:0000027**	Azoospermia
**615842**	SPERMATOGENIC FAILURE 14; SPGF14
**615841**	SPERMATOGENIC FAILURE 13; SPGF13
**615413**	SPERMATOGENIC FAILURE 12; SPGF12
**615234**	ANEMIA, HYPOCHROMIC MICROCYTIC, WITH IRON OVERLOAD 2; AHMIO2
**614897**	HYPOGONADOTROPIC HYPOGONADISM 16 WITH OR WITHOUT ANOSMIA; HH16
**614837**	HYPOGONADOTROPIC HYPOGONADISM 8 WITH OR WITHOUT ANOSMIA; HH8
**613957**	SPERMATOGENIC FAILURE 8; SPGF8
**613724**	LEUKOENCEPHALOPATHY WITH DYSTONIA AND MOTOR NEUROPATHY
**601076**	MULLERIAN DUCT APLASIA, UNILATERAL RENAL AGENESIS, AND CERVICOTHORACIC SOMITE ANOMALIES; MURCS
**415000**	SPERMATOGENIC FAILURE, Y-LINKED, 2; SPGFY2
**400003**	DELETED IN AZOOSPERMIA; DAZ
**312300**	ANDROGEN INSENSITIVITY, PARTIAL; PAIS
**300845**	MOYAMOYA DISEASE 4 WITH SHORT STATURE, HYPERGONADOTROPIC HYPOGONADISM, AND FACIAL DYSMORPHISM; MYMY4
**279000**	YOUNG SYNDROME
**278850**	46,XX SEX REVERSAL 2; SRXX2
**277180**	VAS DEFERENS, CONGENITAL BILATERAL APLASIA OF; CBAVD
**270960**	SPERMATOGENIC FAILURE 4; SPGF4
**241000**	HYPOGONADISM WITH LOW-GRADE MENTAL DEFICIENCY AND MICROCEPHALY
**235200**	HEMOCHROMATOSIS; HFE
**210900**	BLOOM SYNDROME; BLM

- Therefore, user can view entity/term by their Name by **VizMapper** function of Cytoscape

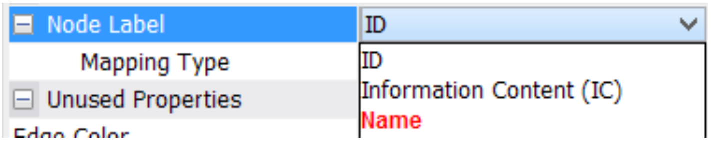

2.4.2 Entity-related Functions

For convenience, Entity-oriented Functions are organized on two tabs:

- **Entity (Set)**
  - This tab includes Functional Similarity Matrix Calculation among selected entities, Enrichment Analysis of selected entities and Visualization.
- **Between Entity Sets**
  - This tab is for calculating functional similarity between entity sets

a. Calculate Functional Similarity Matrix

Step 1: Select **Entity (Set)** tab, then select a set of entities of interest in **Input** tab.

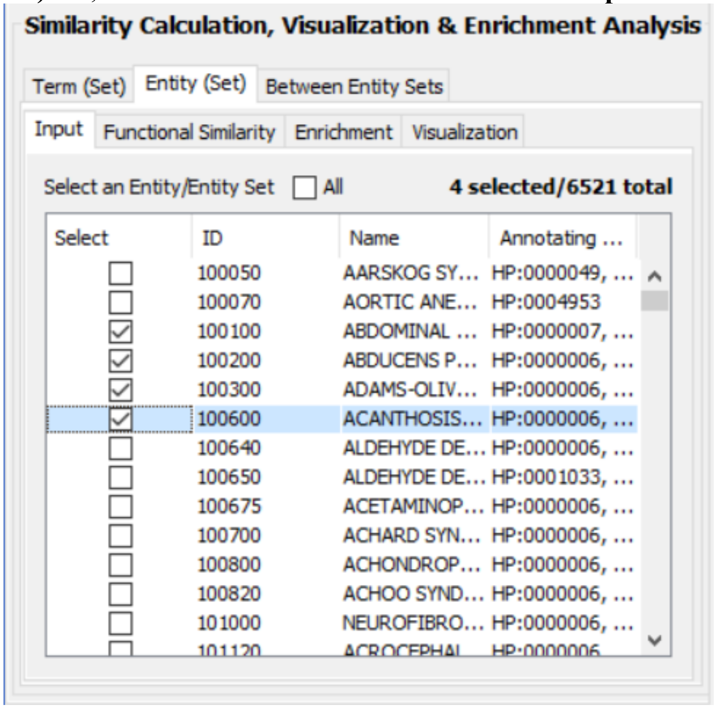

- Note that
  - For *Functional Similarity Calculation* and *Enrichment*: Select at least two entities.

Step 2: Calculate Functional Similarity Matrix

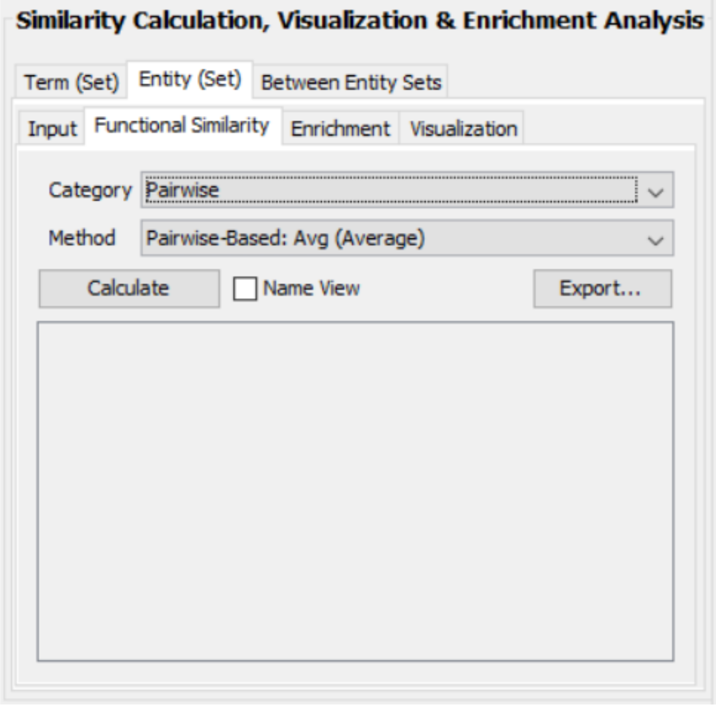

- Select **Category** of methods to calculate functional similarity between two terms (Pesquita, et al., 2009)

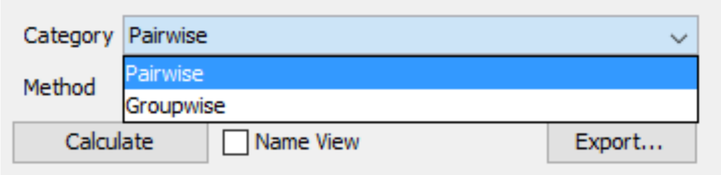

- Select **Method** in the selected **Category**

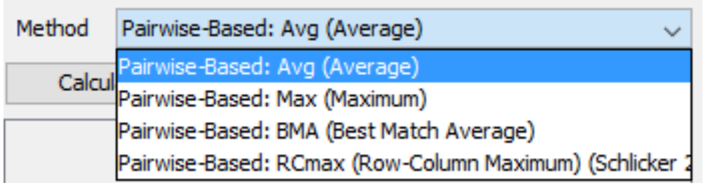

- Note that
  - For **Pair-wise** methods, a pair of terms annotating to each entity must be calculated their semantic similarity based on the selected **Method** which is selected in **Semantic Similarity** tab of **Term (Set)** tab. For example, the selected semantic similarity method is *Node-based: Lin (Lin 1998)*
  - For **Group-wise** methods, users do not need to specify the semantic similarity method for term.

- Click 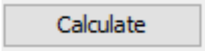 to calculate functional similarity matrix

➔ Result

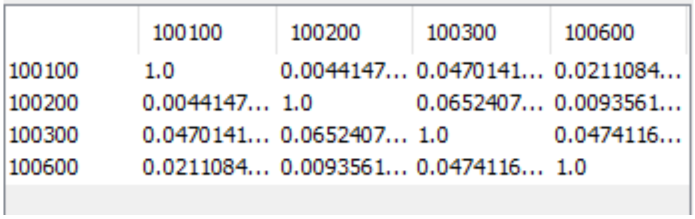

	100100	100200	100300	100600
100100	1.0	0.0044147...	0.0470141...	0.0211084...
100200	0.0044147...	1.0	0.0652407...	0.0093561...
100300	0.0470141...	0.0652407...	1.0	0.0474116...
100600	0.0211084...	0.0093561...	0.0474116...	1.0

Check 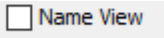 to turn to Entity Name view of the functional similarity matrix

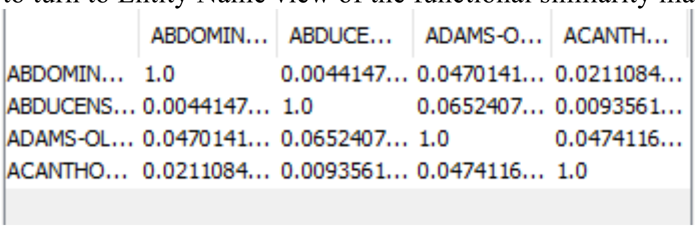

	ABDOMIN...	ABDUCE...	ADAMS-O...	ACANTH...
ABDOMIN...	1.0	0.0044147...	0.0470141...	0.0211084...
ABDUCENS...	0.0044147...	1.0	0.0652407...	0.0093561...
ADAMS-OL...	0.0470141...	0.0652407...	1.0	0.0474116...
ACANTHO...	0.0211084...	0.0093561...	0.0474116...	1.0

- Click 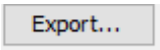

to export to tab delimited-text file

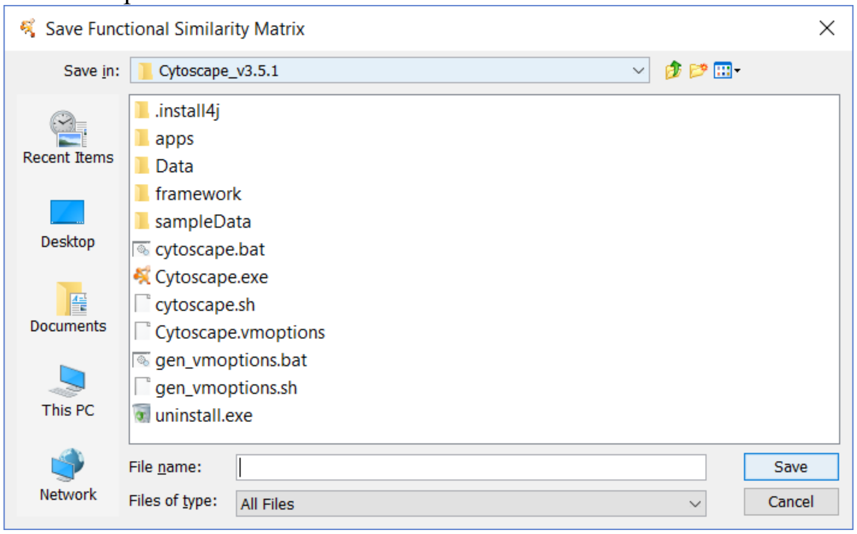

Detail of FunSimMat.txt file

```
ID	ABDOMINAL MUSCLES, ABSENCE OF, WITH URINARY TRACT ABNORMALITY AND CRYPTORCHIDISM	ABDUCENS PALSY	ADAMS-OLIVER SYNDROME 1; AOS1	ACANTHOSIS NIGRICANS
ABDOMINAL MUSCLES, ABSENCE OF, WITH URINARY TRACT ABNORMALITY AND CRYPTORCHIDISM	1.0	0.004414741161548392	0.04701416855323756	0.021108486965106463
ABDUCENS PALSY	0.004414741161548392	1.0	0.0652407231735359	0.009356199289017913
ADAMS-OLIVER SYNDROME 1; AOS1	0.04701416855323756	0.0652407231735359	1.0	0.047411637177936644
ACANTHOSIS NIGRICANS	0.021108486965106463	0.009356199289017913	0.047411637177936644	1.0
```

c. Analyze term enrichment

Step 1: Select **Entity (Set)** tab, then select a set of entities of interest in **Input** tab. For example, four phenotypes with OMIM ID 100100, 100200, 100300, and 100600 are selected.

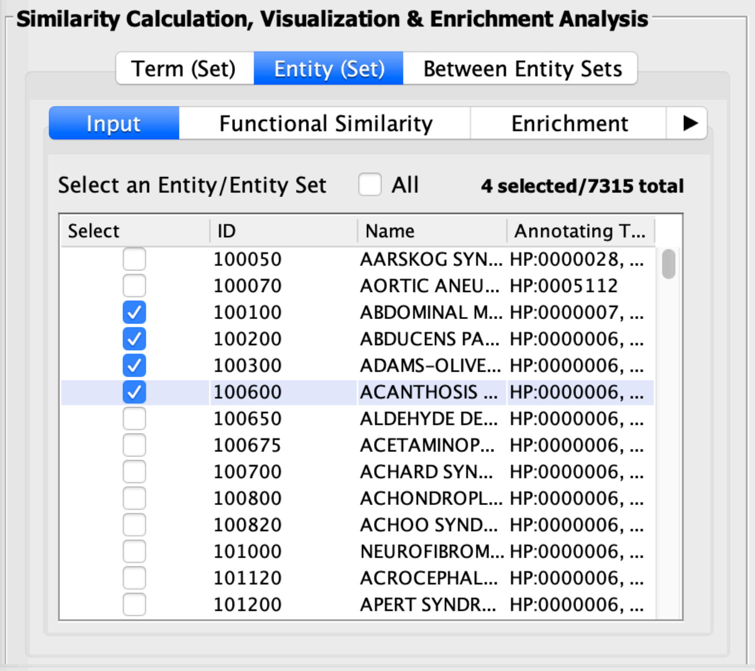

- Note that
  - For *Functional Similarity Calculation* and *Term Enrichment*: Select at least two entities.

Step 2: Select Enrichment tab, then choose methods of Statistical Test and Adjusted P-value

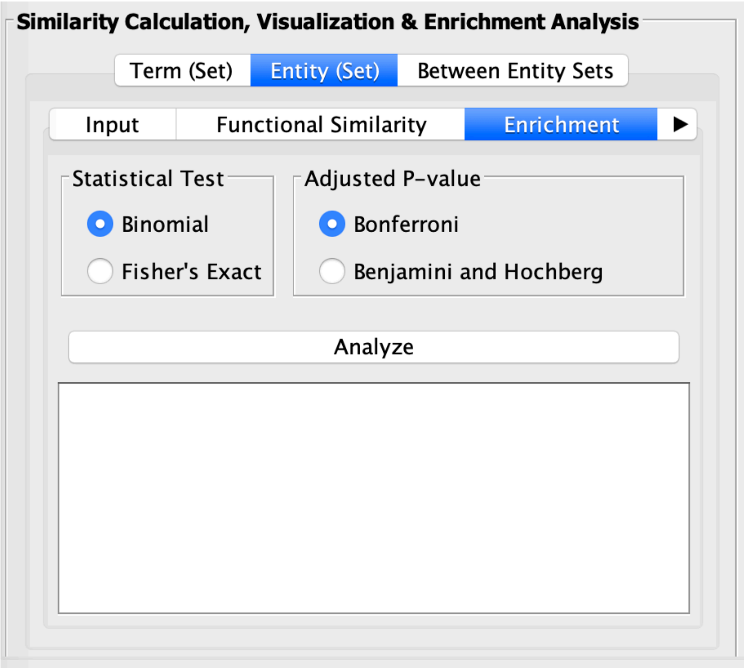

- Note that
  - Enrichment Analysis is performed based on semantic similarity and functional similarty measures, which are selected in **Semantic Similarity** tab in **Term (Set)** tab, and **Fucntional Similarity** tab in **Entity (Set)** tab.
- After that, click 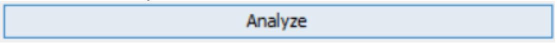

to do Enrichment Analysis. For example, with semantic similarity *Node-based: Resnik (Resnik 1995)* and functional similarity *Pairwise-Based: Avg (Average)*

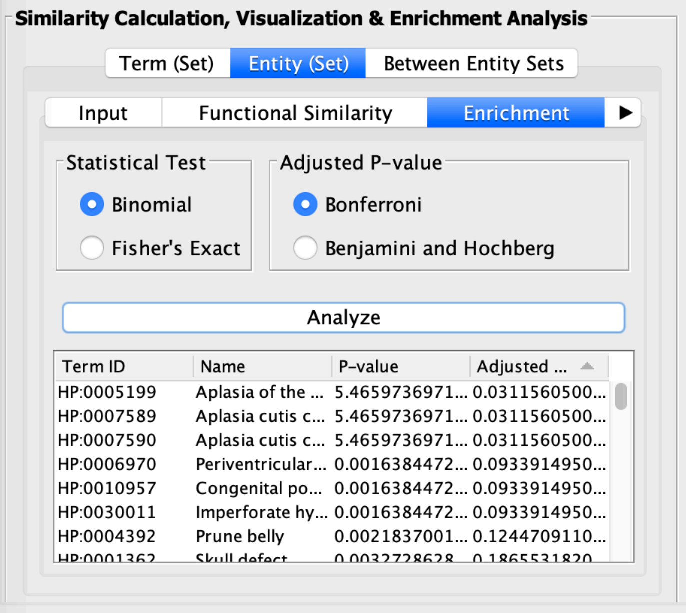

  - Terms having Adjusted P-value ≤ 0.05 (HP: 0005199, HP: 0007589 and HP:0007590) could be of interest and represent functions of the selected set (four phenotypes with OMIM ID 100100, 100200, 100300, and 100600)

d. Visualize terms and annotated entities

<u>*Step 1:*</u> Select **Entity (Set)** tab, then select a set of entities of interest in **Input** tab.

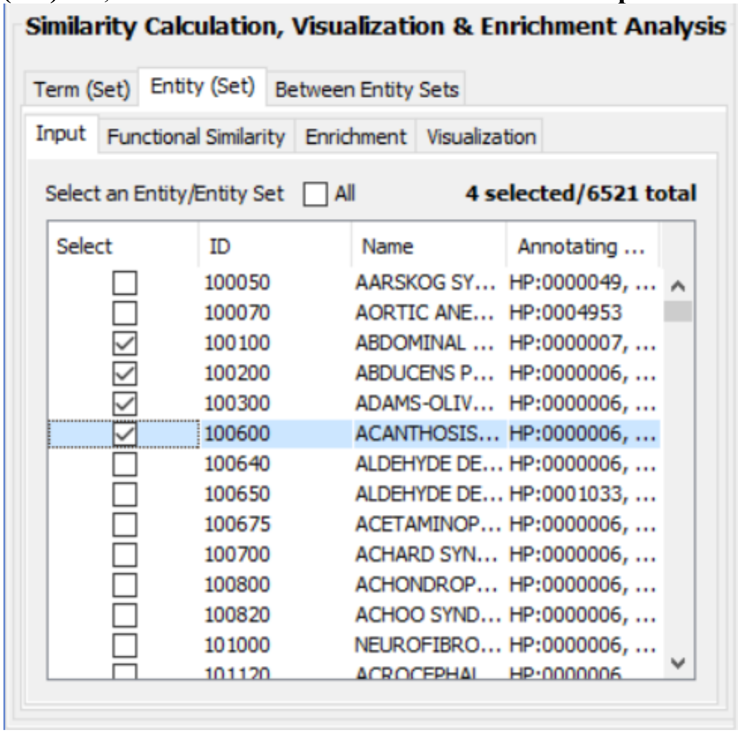

<u>*Step 2:*</u> Visualize terms and annotated entities

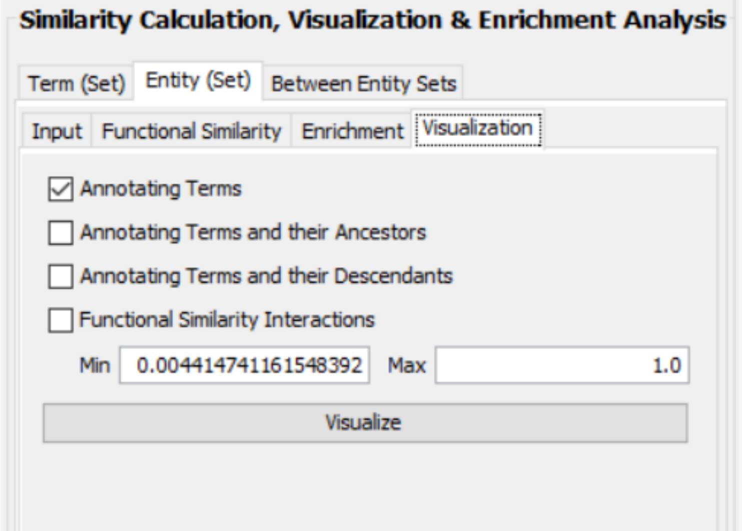

- Select options then click 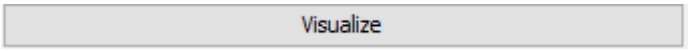

➔ Result

- With **Annotating Terms** (e.g., with 2 entities 100200 and 100600)

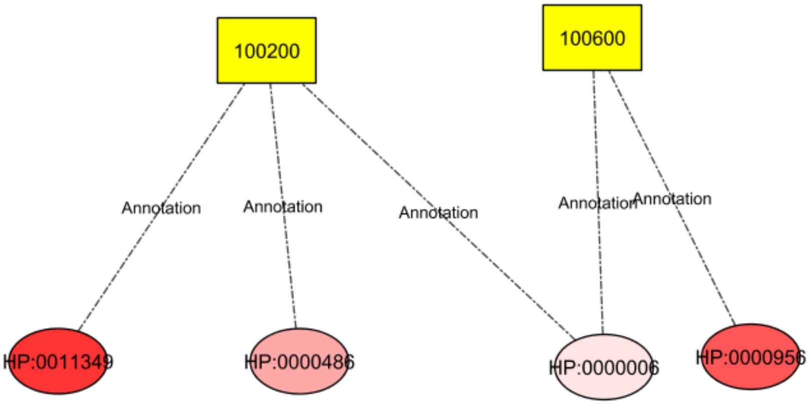

- With **Annotating Terms and Their Ancestors**

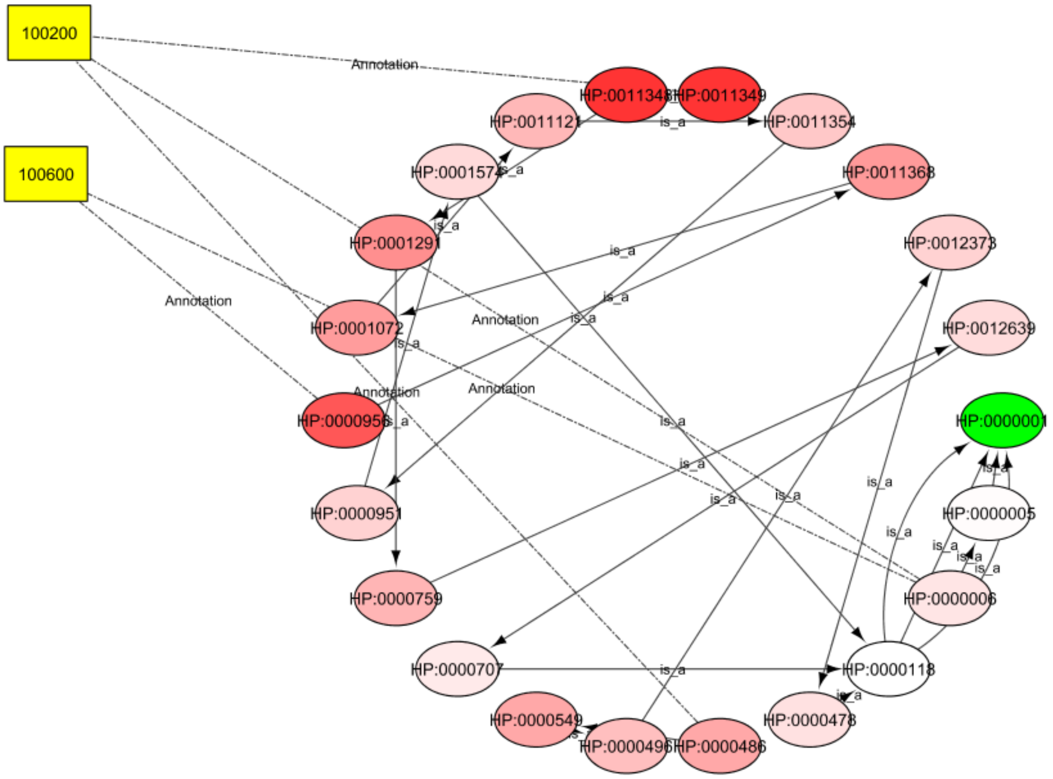

- With **Annotating Terms and Their Descendants**

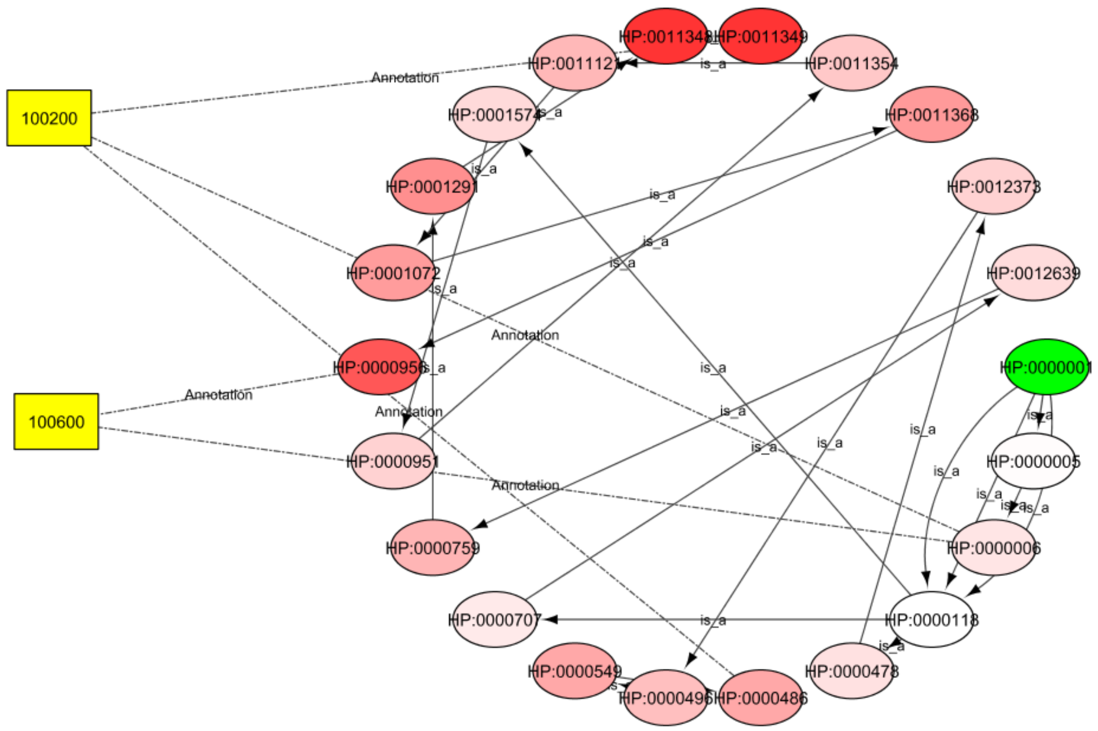

- With **Functional Similarity Interactions**
  - Set thresholds (Min, Max) to visualize functional similarities among selected entities in a form of network

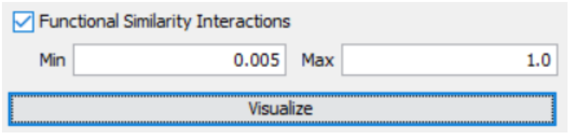

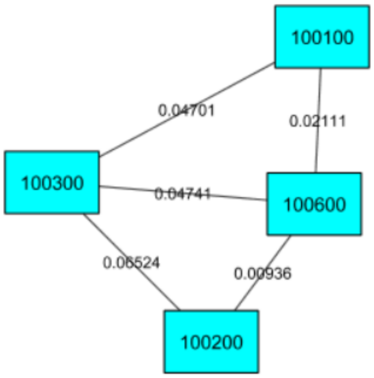

- Note that:
  - To show more detail information of nodes and links in each visualization, users should select **Node Table** and Edge **Table tabs** of Cytoscape.

For example: Detail information of Term and Entity

ID	Information Content (IC)	Name
**100200**		ABDUCENS PALSY
**100600**		ACANTHOSIS NIGRICANS
**HP:0000001**	-0.0	All
**HP:0000005**	0.045010740018377814	Mode of inheritance
**HP:0000006**	0.3869904138502123	Autosomal dominant inheritance
**HP:0000118**	0.005563227857864829	Phenotypic abnormality
**HP:0000478**	0.4552379741481966	Abnormality of the eye
**HP:0000486**	1.2997664475471735	Strabismus
**HP:0000496**	0.9551759029129288	Abnormality of eye movement
**HP:0000549**	1.2958002603295722	Abnormal conjugate eye movement
**HP:0000707**	0.3149020745351841	Abnormality of the nervous system
**HP:0000759**	1.1136104830624403	Abnormal peripheral nervous system morphology
**HP:0000951**	0.6666378759663608	Abnormality of the skin
**HP:0000956**	2.5132842045434782	Acanthosis nigricans
**HP:0001072**	1.4839004268582687	Thickened skin
**HP:0001291**	1.680775291837242	Abnormality of the cranial nerves
**HP:0001574**	0.5525756528549217	Abnormality of the integument
**HP:0011121**	1.0630350962241173	Abnormality of skin morphology
**HP:0011348**	3.036162949823816	Abnormality of the sixth cranial nerve
**HP:0011349**	3.036162949823816	Abducens palsy

Node Attribute Browser | Edge Attribute Browser | Network Attribute Browser

For example: Detail information of Term-Term and Entity-Term relations

ID	interaction
**HP:0011349 (Object-Term) 1002...**	Object-Term
**HP:0000006 (Object-Term) 1006...**	Object-Term
**HP:0000486 (Object-Term) 1002...**	Object-Term
**HP:0000006 (Object-Term) 1002...**	Object-Term
**HP:0000956 (Object-Term) 1006...**	Object-Term
**HP:0001291 (is_a) HP:0000759**	is_a
**HP:0011368 (is_a) HP:0001072**	is_a
**HP:0000006 (is_a) HP:0000005**	is_a
**HP:0000118 (is_a) HP:0000001**	is_a
**HP:0011121 (is_a) HP:0011354**	is_a
**HP:0012639 (is_a) HP:0000707**	is_a
**HP:0000707 (is_a) HP:0000118**	is_a
**HP:0000956 (is_a) HP:0011368**	is_a
**HP:0000549 (is_a) HP:0000496**	is_a
**HP:0011349 (is_a) HP:0011348**	is_a
**HP:0000486 (is_a) HP:0000549**	is_a
**HP:0001574 (is_a) HP:0000118**	is_a
**HP:0000478 (is_a) HP:0000118**	is_a
**HP:0011354 (is_a) HP:0000951**	is_a
**HP:0011348 (is_a) HP:0001291**	is_a

Node Attribute Browser | Edge Attribute Browser | Network A

e. *Assess functional similarity between entity sets*

- Define elements of two sets, then click to calculate the functional similarity between two selected sets. For example, here is the functional similarity between two gene sets as following:

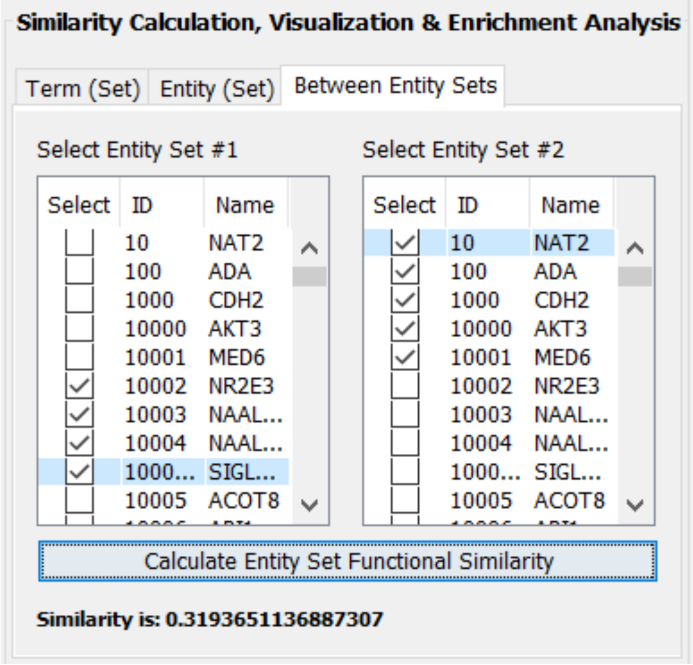

2.5 Utilities

Select Apps → UFO → Utilities…

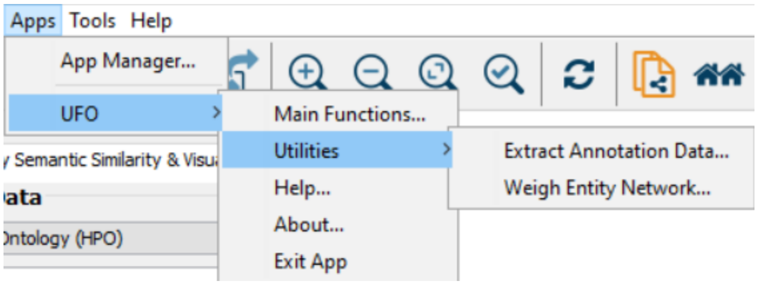

UFO have functions to extract annotation data for genes and phenotypes and to weigh entity network.

2.5.1 Extract annotation data

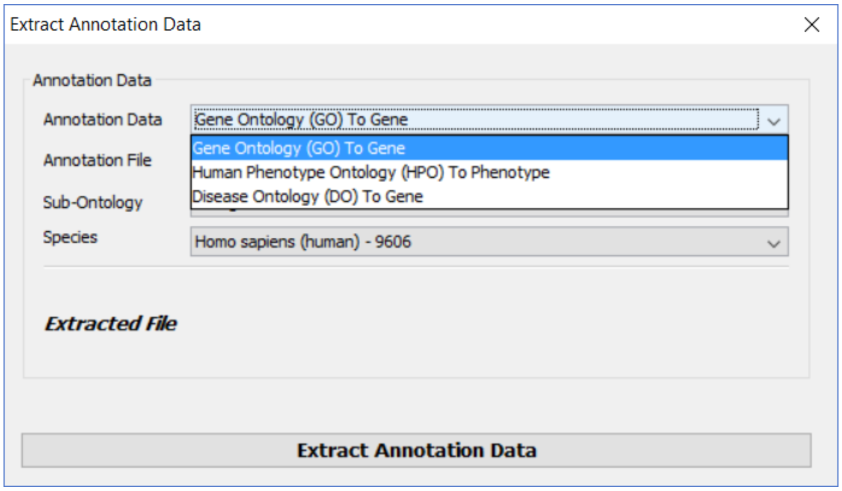

a. Gene Ontology (GO) To Gene

Annotation data of gene by Gene Ontology (gene2go) can be downloaded from NCBI FTP site (ftp.ncbi.nlm.nih.gov/gene/DATA/gene2go.gz), then user can select Sub-Ontology (Biological Process, Cellular Component, and Molecular Functions) and Species to extract annotation data to store in file with format indicated in section **2.1 Prepare Data** (EntityID<tab>OntologyTermID<tab>EvidenceCode)

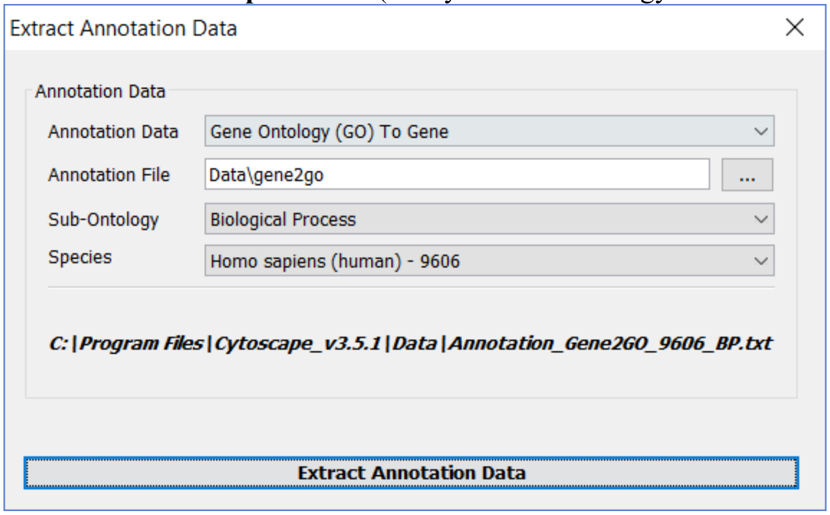

b. *Human Phenotype Ontology (HPO) To Phenotype*

Annotation data of phenotype by Human Phenotype Ontology (phenotype_annotation_hpoteam.tab) can be downloaded from http://www.human-phenotype-ontology.org/(Köhler, et al., 2014).

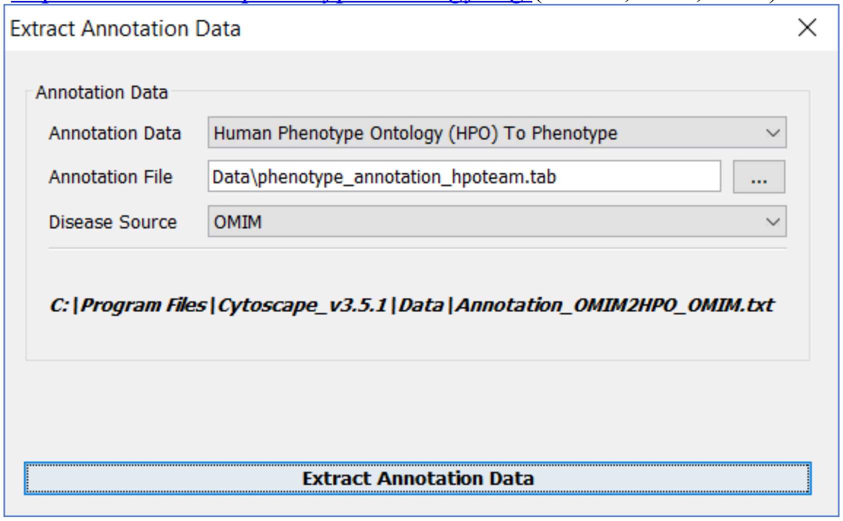

c. *Disease Ontology (DO) To Gene*

Annotation data of phenotype by Human Disease Ontology (IDMappings.rdf) can be downloaded from http://dga.nubic.northwestern.edu/ (Peng, et al., 2013).

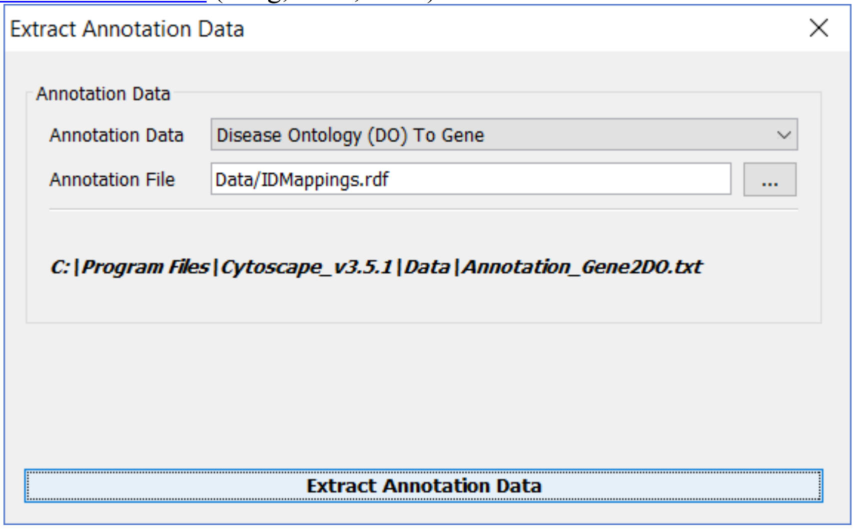

2.5.2 Weigh entity network

The entity network can be a network of genes/proteins, a network of phenotypes. In the study (Le and Kwon, 2013), we used GO-based similarity to weigh protein interaction network to create gene similarity networks using three types of gene ontology i.e., biological process, cellular component and molecular function.

To weigh an entity network

- An entity network must be loaded to Cytoscape (Note that: Network nodes must be identified by the same ID as Entity ID in the used Annotation data). For example:
    - A physical protein interaction network is downloaded from NCBI FTP site (ftp://ftp.ncbi.nlm.nih.gov/gene/GeneRIF/interactions.gz), where proteins are identified by Entrez Gene ID. Then, it is loaded to Cytoscape.

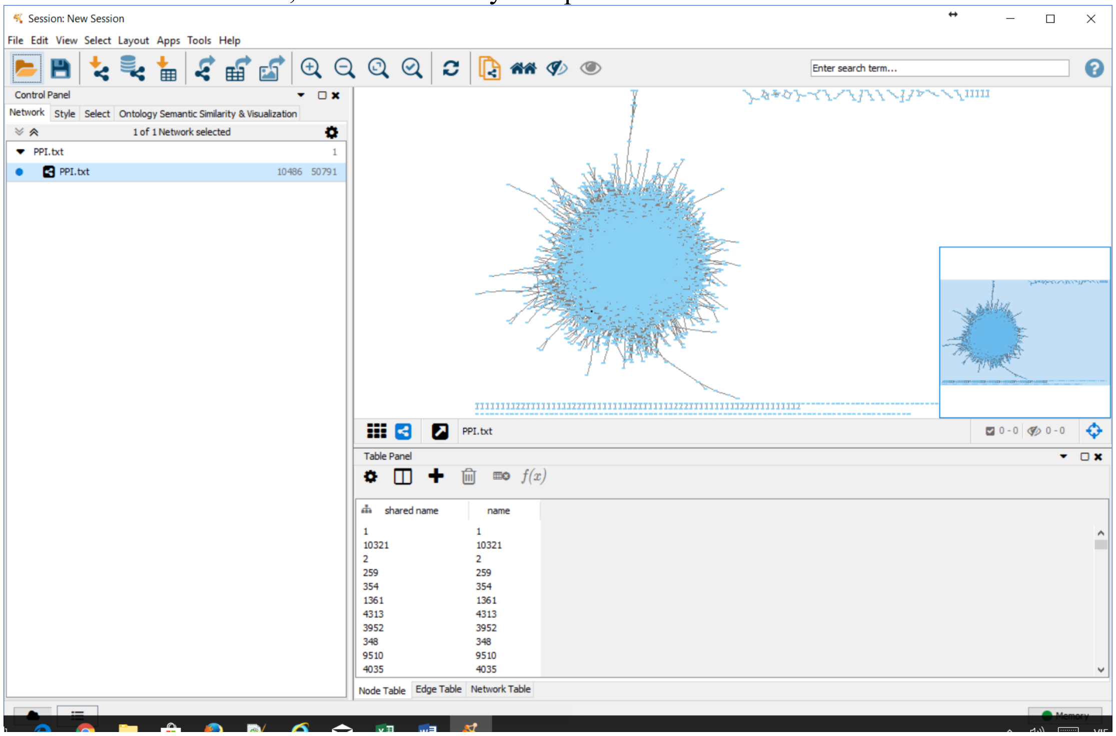

- Then, a suitable ontology and annotation data must be used. In this case, proteins in the loaded network are identified by Entrez Gene ID, therefore, Gene Ontology and Annotation must be used. For example, sub-ontology **Gene Ontology (GO) – Biological Process** and annotation **Gene Ontology (GO) To Gene** are used by clicking on **Load & Preparing Data**

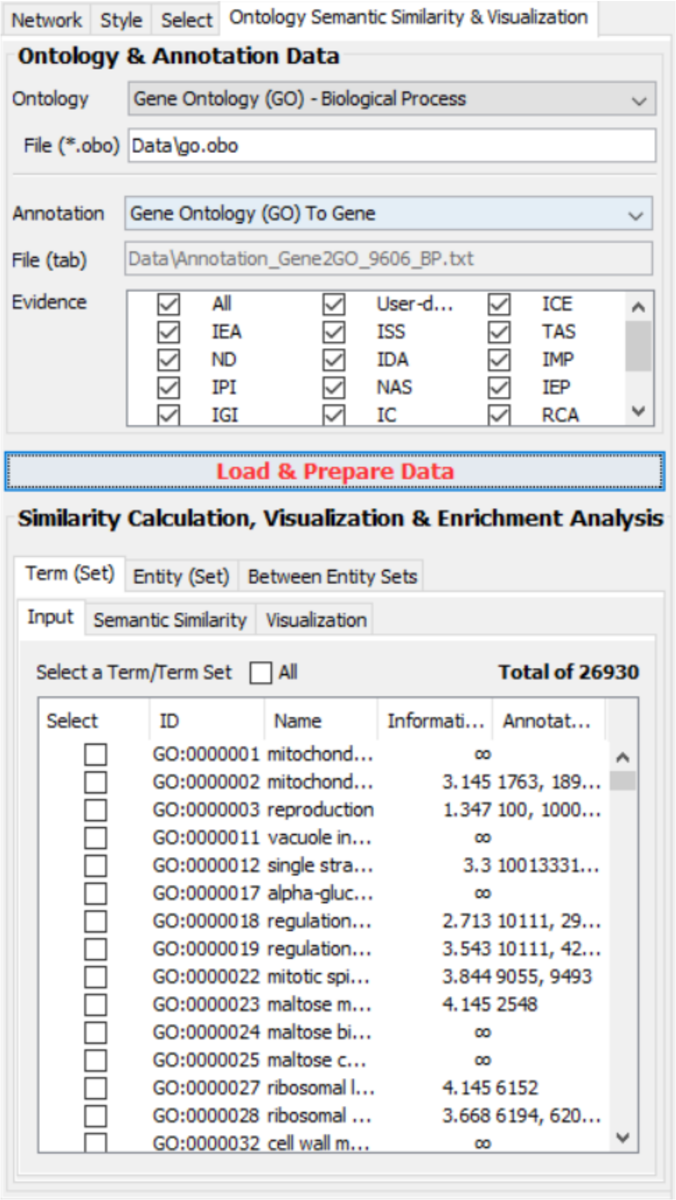

- After that, a semantic similarity measure between terms (GO) and a functional similarity measure between entities (Gene) must be specified. For example, semantic similarity measure *Node-based: Resnik (Resnik 1995)* and functional similarity measure *Pairwise-Based: Avg (Average)* are specified.

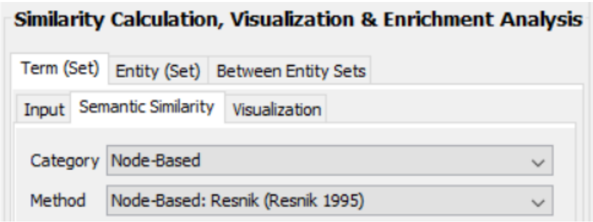

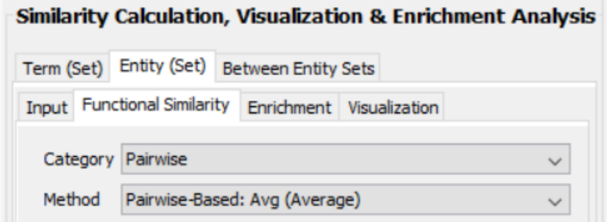

- Select some interactions in the loaded network to be weighted

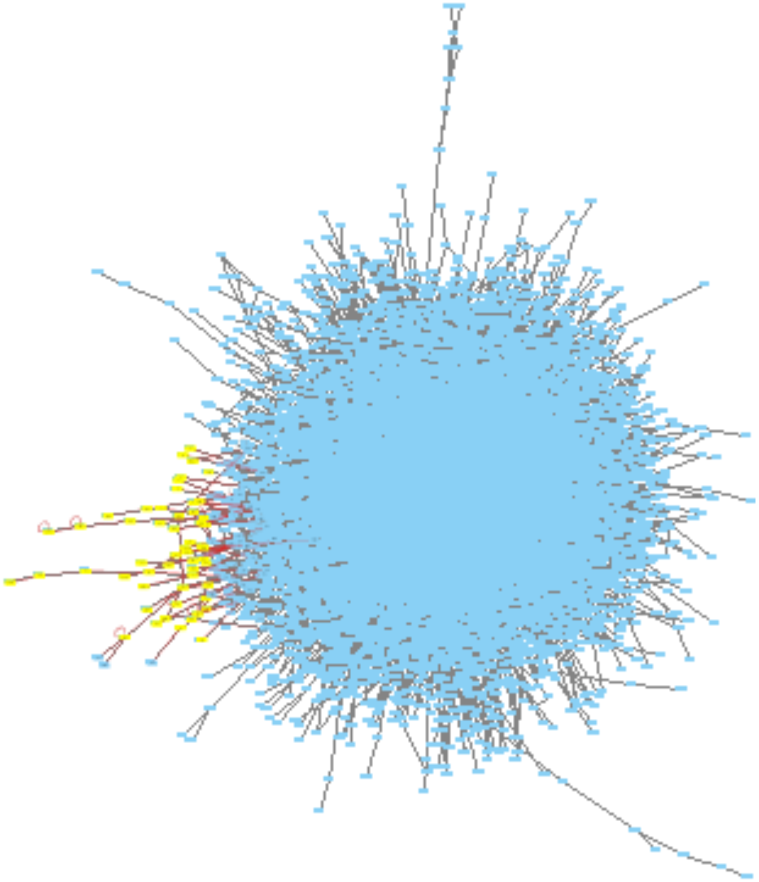

- Go to **Apps → UFO → Utilities… → Weigh Entity Network…**, select the loaded network and **Selected Interactions** option to weigh

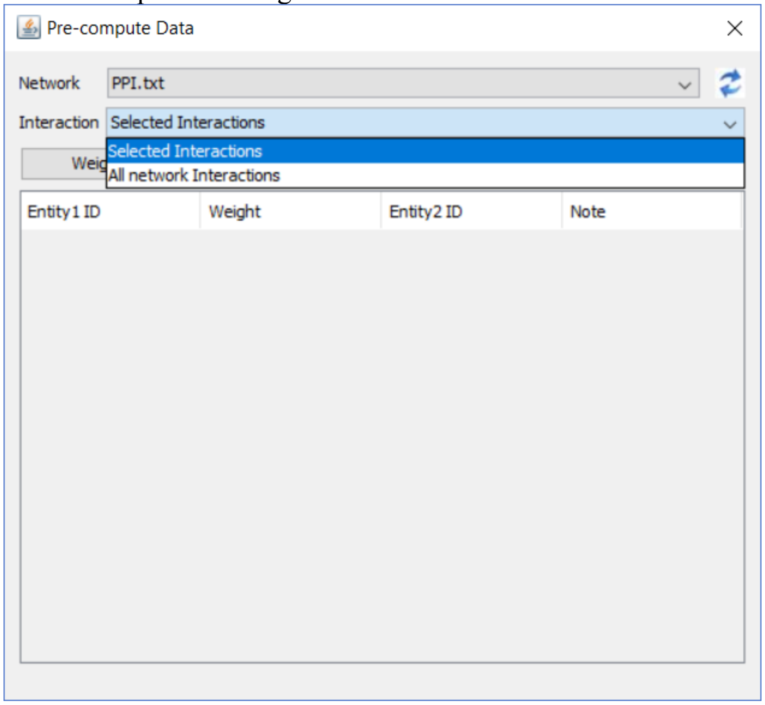

- Click 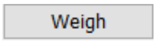 to weigh selected interactions

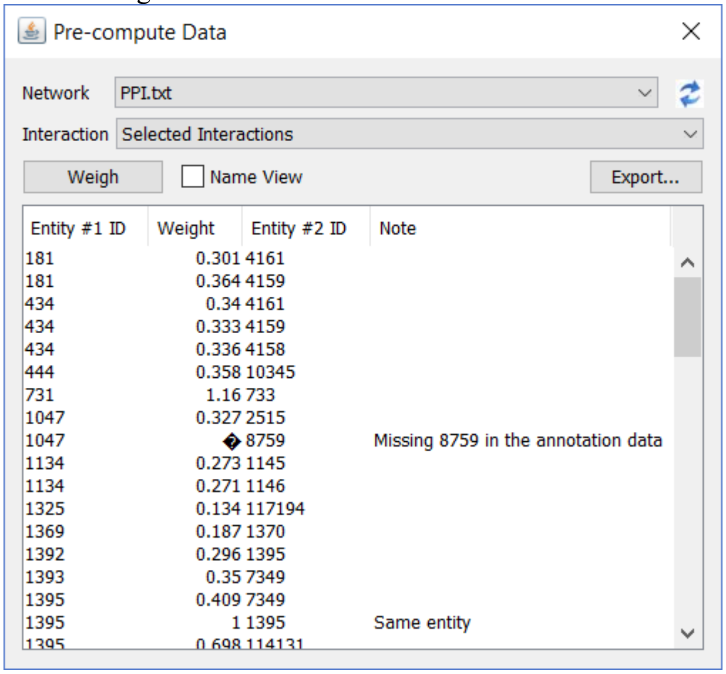

- If option **All network interactions** is selected, then all interactions in the loaded network will be weighted.

3 CASE STUDY

Comparing different similarity measures could help researchers choose the most appropriate measure for their biological application (Mazandu and Mulder, 2014). Pesquita et al., assessed similarity between genes based on gene ontology (Pesquita, et al., 2009). Since UFO provides batch calculation of similarity, thus we recently estimated the similarity of 9,221,365 pairs of 4,295 phenotypes based on HPO using 47 between-entity similarity measures (Le, et al., 2016). This provides guidelines for future studies which need to choose the most approximate semantic similarity method to assess phenotypic similarity between diseases. In addition, we have employed UFO for constructing gene and protein complex similarity network using GO for predicting disease-associated genes (Le and Kwon, 2013) and protein complexes (Le, 2015), respectively; disease similarity network using HPO for predicting disease-associated genes (Le and Dang, 2016); and disease similarity network using DO and HPO for predicting disease-associated lncRNAs (Le and Dao, 2018) (See more detail in Additional file 3).

3.1 Assessing human disease phenotype similarity based on ontology

The following figures (adapted from our previous study (Le, et al., 2016)) shows comparison for pairwise between-term measures using Pearson and Spearman correlation coefficients.

For pair-wise methods, the results showed that, for both types of correlation, the largest correlation coefficient is 0.58 for BMA (Azuaje, et al., 2005; Couto, et al., 2005) and Max pairwise between-entity similarity with Rel (Schlicker, et al., 2006) and Wu2005 (Wu, et al., 2005) between-term measures, respectively (Le, et al., 2016) (see the red-dash box of the following figures).

a

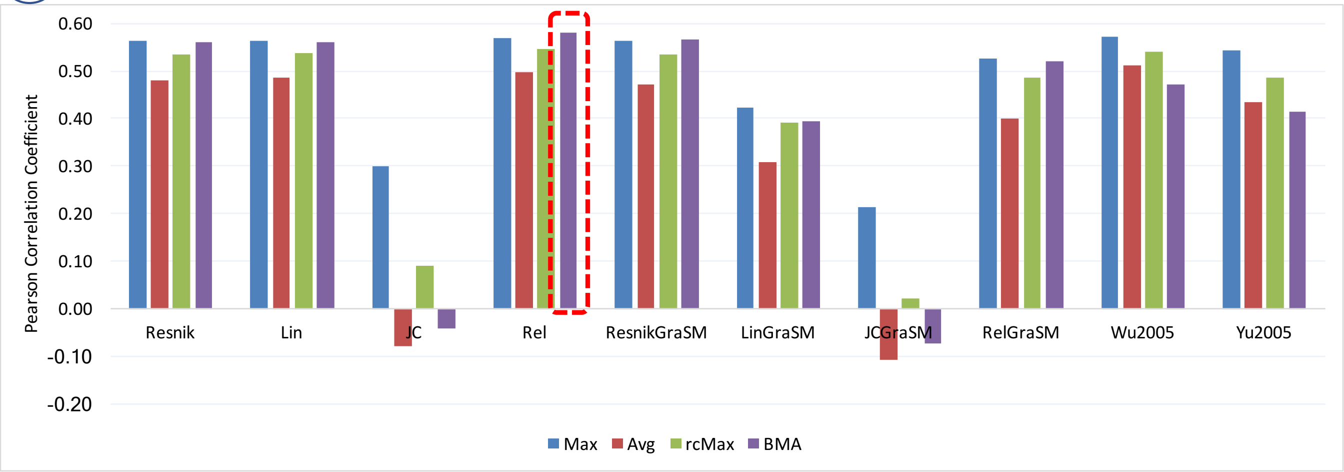

b

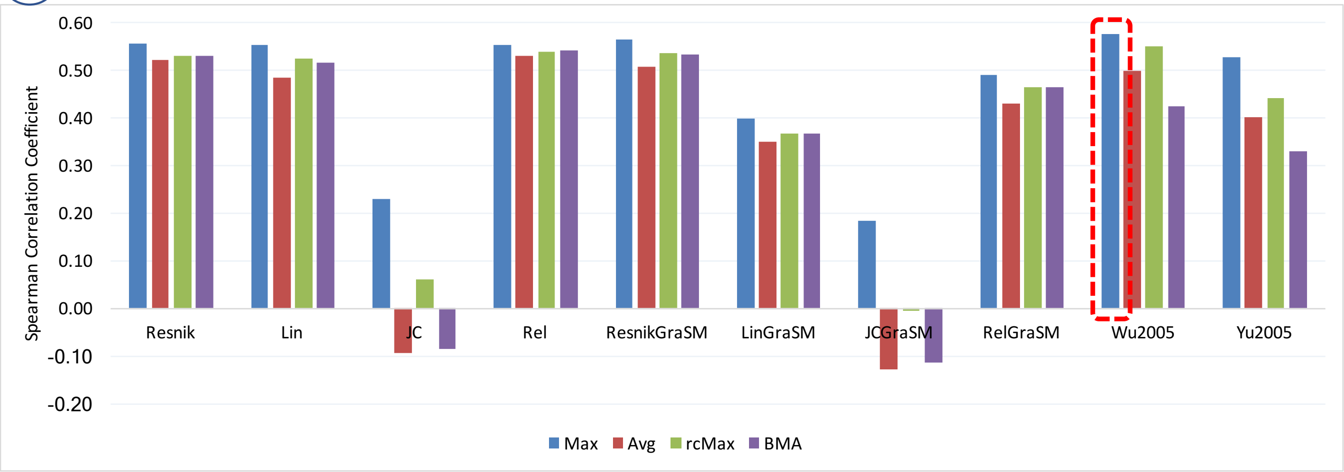

For group-wise methods, best measures were LP between-entity (Gentleman, 2005) for both correlation methods and TO between-entity method for Spearman correlation (Le, et al., 2016) (see the red-dash box of the following figures).

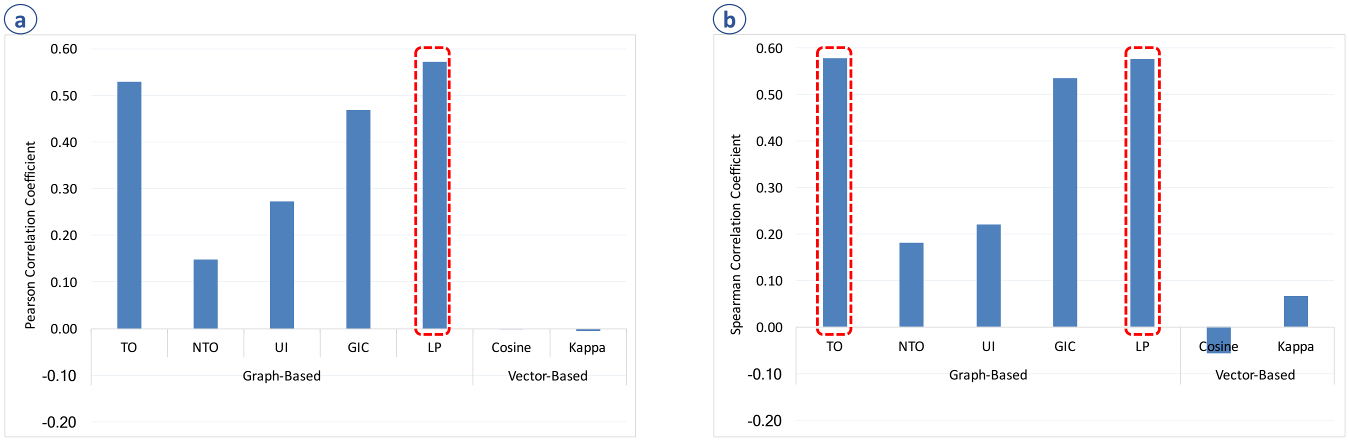

3.2 Construct gene and protein complex similarity network using GO for predicting disease-associated genes and protein complexes

The following figure illustrates the proposed method in (Le and Kwon, 2013). In the study, we weigh an unweighted protein interaction using three sub-gene ontologies (i.e., biological process, cellular component and molecular function) to create three weighted protein similarity networks as following figures:

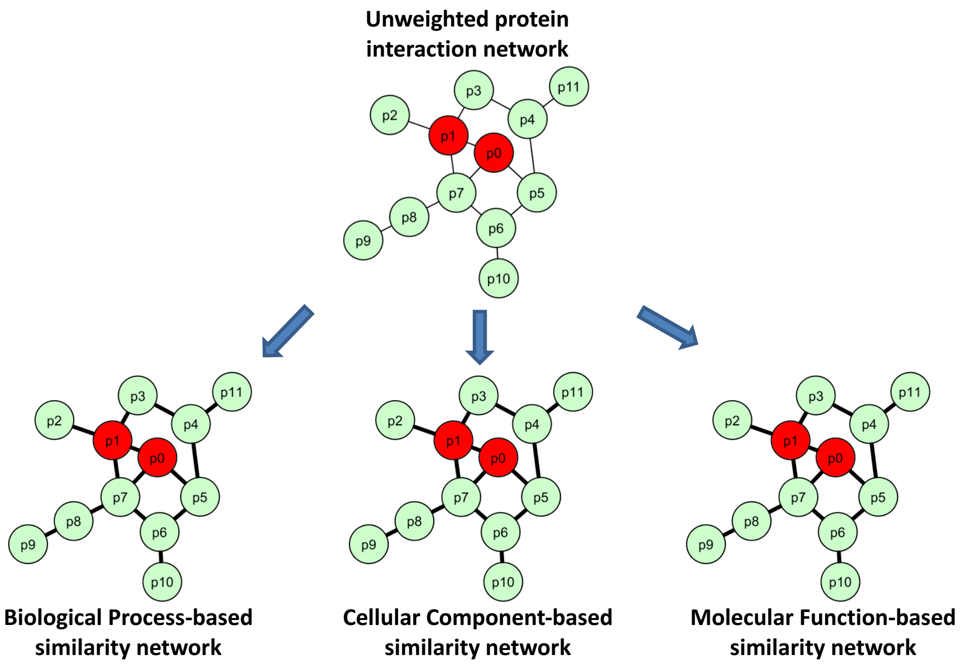

The following figure (adapted from our previous study (Le, 2015)) illustrates how a functional similarity interaction between two protein complexes was created. This interaction was created based on shared GO terms which are used to annotate protein elements in protein complexes (see the red-dash box in the following figures).

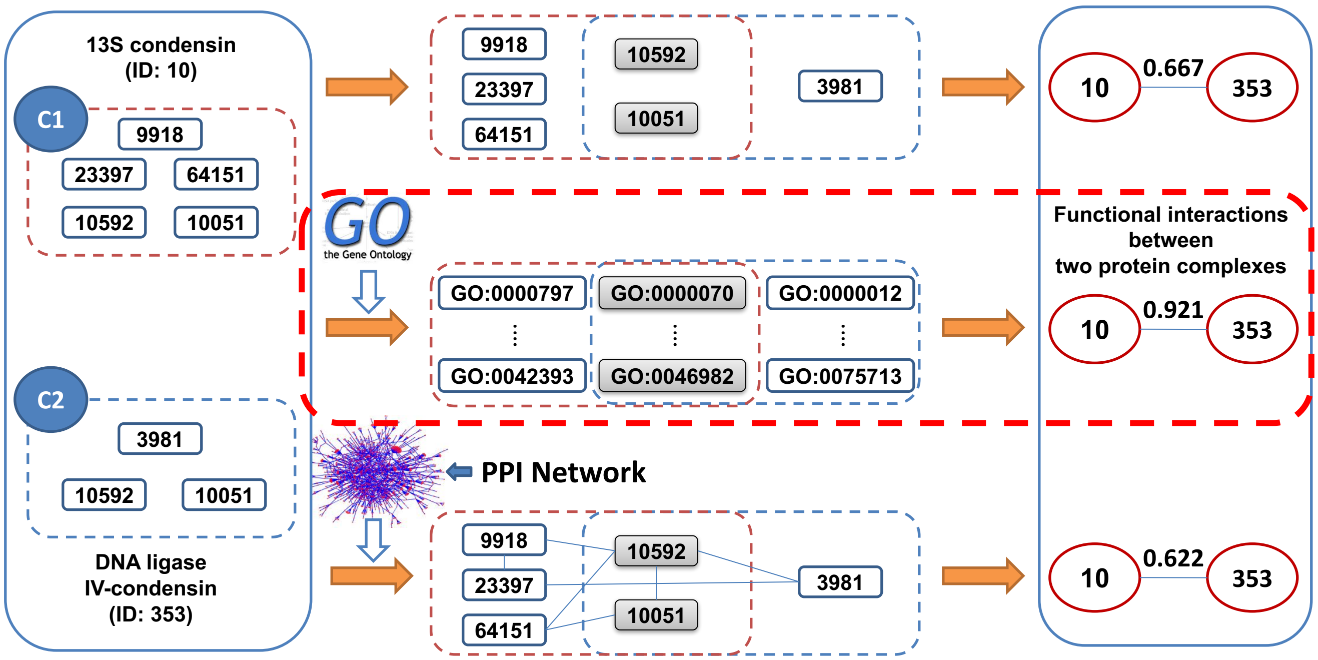

3.3 Construct disease similarity network using HPO and DO for predicting disease-associated genes and lncRNAs

The following figure (adapted from our previous study (Le and Dang, 2016)) illustrates the method proposed in the study. In the study, we calculated semantic similarity for every pair of disease phenotypes using HPO (see the red-dash box in the following figure), then a threshold was set to create a disease similarity network.

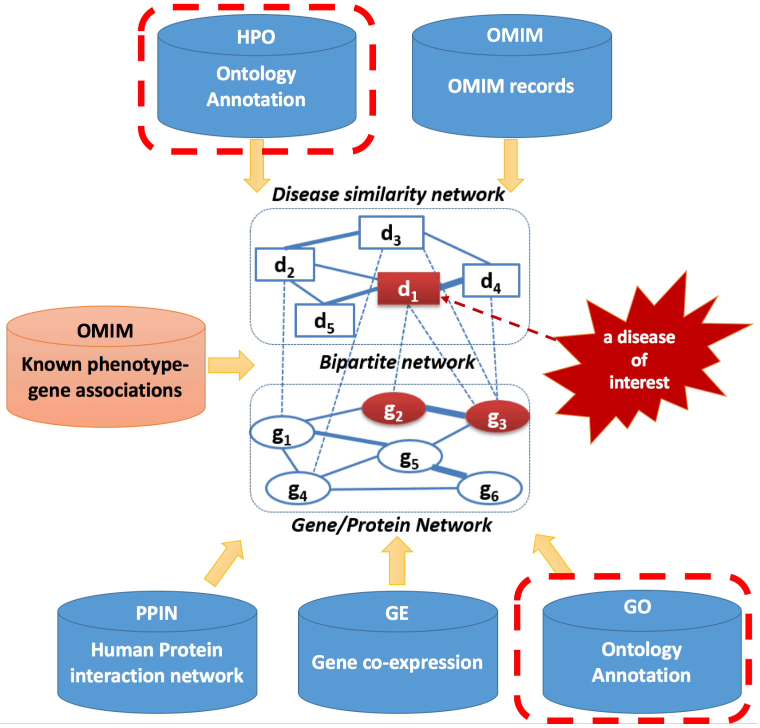

The red-dash box in the following figure (adapted from our previous study (Le and Dao, 2018)) shows how to estimate a similarity between two diseases using HPO and DO.

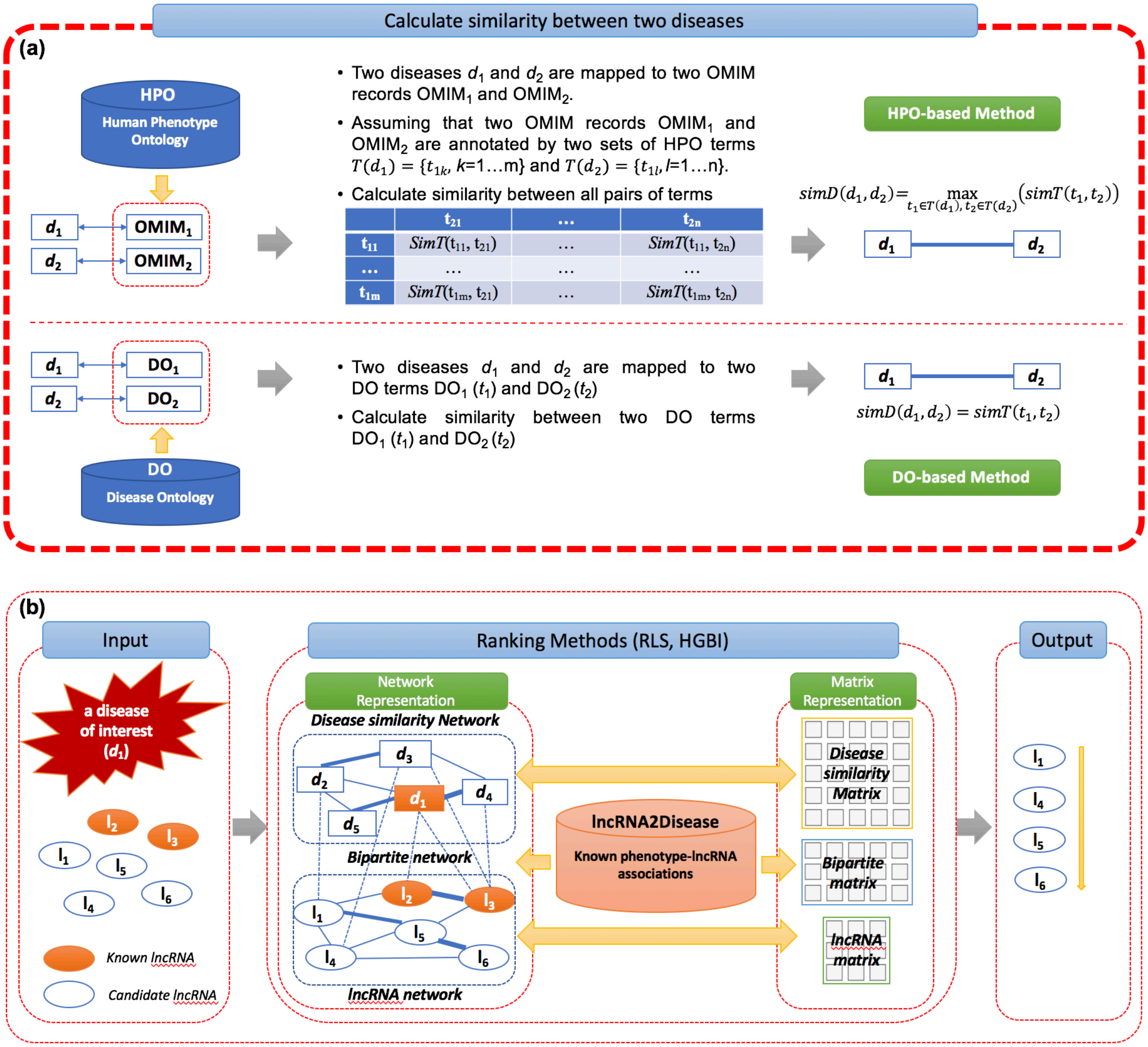
Calculate similarity between two diseases
(a)
HPO
Human Phenotype Ontology
d1
d2
OMIM1
OMIM2
• Two diseases d1 and d2 are mapped to two OMIM records OMIM1 and OMIM2.
• Assuming that two OMIM records OMIM1 and OMIM2 are annotated by two sets of HPO terms T(d1) = {t1k, k=1…m} and T(d2) = {t1l, l=1…n}.
• Calculate similarity between all pairs of terms
t21 … t2n
t11 SimT(t11, t21) … SimT(t11, t2n)
… … … …
t1m SimT(t1m, t21) … SimT(t1m, t2n)
HPO-based Method
simD(d1, d2) = max t1∈T(d1), t2∈T(d2) (simT(t1, t2))
DO1
DO2
DO
Disease Ontology
• Two diseases d1 and d2 are mapped to two DO terms DO1 (t1) and DO2 (t2)
• Calculate similarity between two DO terms DO1 (t1) and DO2 (t2)
simD(d1, d2) = simT(t1, t2)
DO-based Method
(b)
Input
a disease of interest (d1)
Known lncRNA
Candidate lncRNA
Ranking Methods (RLS, HGBI)
Network Representation
Disease similarity Network
Bipartite network
lncRNA network
lncRNA2Disease
Known phenotype-lncRNA associations
Matrix Representation
Disease similarity Matrix
Bipartite matrix
lncRNA matrix
Output

4 REFERENCE

Azuaje, F., Wang, H. and Bodenreider, O. Ontology-driven similarity approaches to supporting gene func-tional assessment. *Proc. Of The Eighth Annual Bio-Ontologies Meeting* 2005;Michigan, 25 June.

Couto, F., Silva, M.r. and Coutinho, P. Semantic similarity over the gene ontology: family correlation and selecting disjunctive ancestors. In, *CIKM '05: Proceedings of the 14th ACM international conference on Information and knowledge management*. Bremen, Germany: ACM; 2005. p. 343-344.

Gentleman, R. Visualizing and distances using GO. *URL* http://www/. *bioconductor. org/docs/vignettes. html* 2005;38.

Köhler, S.*, et al.* The Human Phenotype Ontology project: linking molecular biology and disease through phenotype data. *Nucleic acids research* 2014;42(D1):D966-D974.

Le, D.-H. A novel method for identifying disease associated protein complexes based on functional similarity protein complex networks. *Algorithms for Molecular Biology* 2015;10(1):14.

Le, D.-H. and Dang, V.-T. Ontology-based disease similarity network for disease gene prediction. *Vietnam Journal of Computer Science* 2016:1-9.

Le, D.-H. and Dao, L.T.M. Annotating Diseases Using Human Phenotype Ontology Improves Prediction of Disease-Associated Long Non-coding RNAs. *Journal of Molecular Biology* 2018;430(15):2219-2230.

Le, D.-H. and Kwon, Y.-K. Neighbor-favoring weight reinforcement to improve random walk-based disease gene prioritization. *Computational Biology and Chemistry* 2013;44(0):1-8.

Le, D.-H., Pham, B.-S. and Dao, A.-M. Assessing human disease phenotype similarity based on ontology. In, *RIVF 2016*. Hanoi: IEEE; 2016. p. 211-216.

Mazandu, G.K. and Mulder, N.J. Information Content-Based Gene Ontology Functional Similarity Measures: Which One to Use for a Given Biological Data Type? *PLOS ONE* 2014;9(12):e113859.

Peng, K.*, et al.* The disease and gene annotations (DGA): an annotation resource for human disease. *Nucleic acids research* 2013;41(D1):D553-D560.

Pesquita, C.*, et al.* Semantic Similarity in Biomedical Ontologies. *PLoS Comput Biol* 2009;5(7):e1000443.

Schlicker, A.*, et al.* A new measure for functional similarity of gene products based on Gene Ontology. *BMC Bioinformatics* 2006;7(1):302.

Wu, H.*, et al.* Prediction of functional modules based on comparative genome analysis and Gene Ontology application. *Nucleic acids research* 2005;33(9):2822-2837.
